# HPLC-DAD-ESI-QTOF-MS Determination of Bioactive Compounds and Antioxidant Activity Comparison of the Hydroalcoholic and Water Extracts from Two *Helichrysum italicum* Species

**DOI:** 10.3390/metabo10100403

**Published:** 2020-10-12

**Authors:** Katja Kramberger, Darja Barlič-Maganja, Dunja Bandelj, Alenka Baruca Arbeiter, Kelly Peeters, Ana Miklavčič Višnjevec, Zala Jenko Pražnikar

**Affiliations:** 1 Faculty of Health Sciences, University of Primorska, 6310 Izola, Slovenia; katja.kramberger@fvz.upr.si (K.K.); darja.maganja@fvz.upr.si (D.B.-M.); 2 Faculty of Medicine, University of Ljubljana, 1000 Ljubljana, Slovenia; 3 Faculty of Mathematics, Natural Sciences and Information Technologies, University of Primorska, 6000 Koper, Slovenia; dunja.bandelj@upr.si (D.B.); alenka.arbeiter@upr.si (A.B.A.); 4 InnoRenew CoE, 6310 Izola, Slovenia; kelly.peeters@innorenew.eu; 5 Andrej Marušič Institute, University of Primorska, 6000 Koper, Slovenia

**Keywords:** *H. italicum*, plant extracts, phenolic compounds, antioxidant activity, HPLC-DAD-ESI-QTOF-MS

## Abstract

Mediterranean plant *Helichrysum italicum* represents a rich source of versatile bioactive compounds with potential benefits for human health. Despite extensive research on the plant’s active constituents, little attention has yet been paid to characterizing the relationship between its intra-specific genetic diversity and metabolite profile. The study aimed to determine metabolic profile of *H. italicum* ssp. *italicum* (*HII*) and ssp. *tyrrhenicum* (*HIT*) cultivated on the experimental plantation in Slovenia and to compare the chemical composition of extracts regarding the solvent extraction process. Extracts were prepared upon conventional extract preparation procedures: maceration with 50% methanol or ethanol and cold or hot water infusion and analyzed using High Performance Liquid Chromatography-Diode Array Detection-Electrospray Ionization-Quadrupole Time-of-Flight-Mass Spectrometry (HPLC-DAD-ESI-QTOF-MS). One hundred compounds were identified in the samples, among them several isomers and derivatives were reported for the first time, while caffeoylquinic acids and pyrones were the most abundant. Semi-quantitative comparison revealed that the extraction procedure had a greater impact on the chemical profile than genetic variability. All *HIT* extracts showed a higher total phenolic content compared to *HII*, while the antioxidant potential evaluated by 1,1-diphenyl-2-picrylhydrazil test was not proportionally higher. In addition, hot water extracts proved to be comparably active as alcoholic ones, confirming high commercial potential of *Helichrysum italicum* as herbal functional beverages.

## 1. Introduction

The plant *Helichrysum italicum* (Roth) G. Don is a perennial subshrub characteristic to the Mediterranean region. The genus *Helichrysum* Mill. (Asteraceae) is very complex, as it comprises cca. 500–600 species, which are geographically distributed also beyond the Mediterranean basin and thus diverse with respect to both phenotype and metabolite profile. *Helichrysum italicum* species itself differ in terms of morphological features, genetic variation and geographical distribution and are further divided into four subspecies (ssp.): *italicum*, *microphyllum*, *siculum* and *tyrrhenicum*. However, their correct taxonomic assignment and clear differentiation are sometimes difficult due to their phenotypic plasticity and existence of intermediates generated with spontaneous hybridization in areas where the subspecies overlap [1,2]. The plant naturally thrives in dry, sandy areas but is also extensively cultivated in several Mediterranean countries due to the high demand for its essential oil by the perfume and cosmetic industry [3]. Namely, its yellow fade-resistant inflorescences are a treasury of bioactive secondary metabolites that result from the plants adaptation to this challenging environment. Apart from volatile terpenes present in essential oils, *H. italicum* is also very rich in phenolic compounds, which are recognized as potential health promoting agents due to antioxidant properties they exert and their probable role in the prevention of various diseases associated with oxidative stress, such as cancer, cardiovascular and neurodegenerative diseases [4]. The health-beneficial potential of *H. italicum* has been reported in ethnopharmacological surveys and supported by numerous in vitro and in vivo experiments [5]. Despite the extensive research, little attention has been devoted to characterizing the relationship between its intra-specific genetic diversity and either growing environment or metabolite profile [6].

In addition to genetic and phenotypic differences, the type and concentration of herbal components and consequently their therapeutic effect is highly dependent on the extraction method used [5]. Biologically active compounds isolated from *H. italicum* ssp. *italicum* and *microphyllum* with regard to isolation procedure have already been summarized by Maksimović et al. [7]. Briefly, phenolic compounds previously reported in *H. italicum* comprise following chemical classes of structurally diverse substances: phenolic acids (hydroxybenzoic and hydroxycinnamic acids), hydroxycinnamic esters, coumarins, flavonoids (flavones, flavonols, flavanones, flavanols), flavonoid ethers, esters and glycosides, acetophenones as well as associates of those classes [7]. Since structures of the identified compounds have already been elucidated by spectroscopic methods, the chemical composition of extracts can be routinely investigated using chromatographic methods. Due to the complexity of the plant samples, a high mass resolution and accuracy, sensitivity as well as sophisticated data analysis software are needed to successfully perform phytochemical screening. A configuration, which is appropriate for phenolic compounds identification and fulfils above-mentioned requirements, is liquid chromatography quadrupole time-of-flight mass spectrometry (LC-QTOF-MS). While there are numerous studies investigating essential oil composition of *H. italicum* (reviewed by Maksimovic et al. [7]), comprehensive chromatographic studies performed on crude solvent extracts are rather scarce. Even less investigated are water-based preparations, which are typically used in traditional medicine. An exception is the research by Pereira et al. [8] who investigated the chemical profile of infusions and decoctions of *H. italicum* ssp. *picardii*, growing in Portugal, in comparison to the commonly consumed tisanes of green tea and rooibos.

The aim of the study was to determine the metabolic profile of two different *H. italicum* subspecies (*italicum* and *tyrrhenicum*) cultivated on an experimental plantation in Slovenian Istria, as well as to compare the chemical composition of extracts in relation to the solvent extraction procedure. As the plants were grown and harvested under the same conditions, the environmental impact on differences in their metabolic profile was thus eliminated. The extracts were prepared upon conventional extract preparation procedures: maceration with 50 % methanol or ethanol and cold or hot water infusion [9]. To the best of our knowledge this is the first study comparing composition of crude hydroalcoholic and water extracts of two different *H. italicum* subspecies using High Performance Liquid Chromatography-Diode Array Detection-Electrospray Ionization-Quadrupole Time-of-Flight-Mass Spectrometry (HPLC-DAD-ESI-QTOF-MS). Additionally, antioxidant properties of extracts were evaluated using a 1,1-diphenyl-2-picrylhydrazyl (DPPH) test and were correlated with the total phenolic content.

## 2. Results and Discussion

### 2.1. Qualitative Analysis

After interpretation of the fragmentation pattern from the collected mass spectra of the *H. italicum* ssp. *italicum* (*HII*) and *tyrrhenicum* (*HIT*) samples (methanol:water (1:1) extracts—MWEs, ethanol:water (1:1) extracts—EWEs, hot water extracts—HWEs and cold water extracts—CWEs), one hundred phenolic compounds were identified. Of these, five compounds could be identified unambiguously with a reference standard and seventy-five could be identified tentatively by matching with published MS/MS spectra. Nine compounds were only partially identified as a derivative of a known compound, according to characteristic fragments present in the spectra. For the remaining eleven compounds, without reference spectra available, identification is less confident, although, supported by accurate mass and isotope pattern match and additionally computed Molecular Structure Correlator (MSC) scores. Detailed MS/MS information of the identified compounds is available in Appendix A, Table A1. In Table A1, compounds are listed in order of elution from the column and numbered accordingly. The same numbering system is maintained throughout the text.

**Figure 1:**
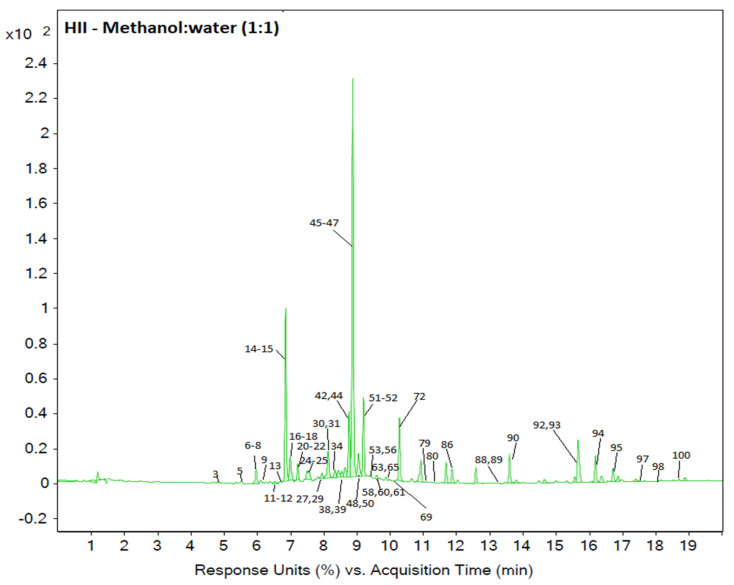
Representative DAD chromatogram (280 nm) of a *H. italicum* ssp. *italicum* (*HII*) sample with peak annotations representing identified compounds. Numbers correspond to each identified compound, listed in Appendix A, Table A1.

LC-ESI-QTOF-MS chromatograms (available in the Supplementary file, Figure S1) of *H. italicum* samples were relatively complex, containing peaks of several hydroxycinnamic and hydroxybenzoic acids, flavonoids, coumarins, pyrones as well as other chemical classes, such as isobenzofuranones, neolignans and acetophenones. The majority of the targeted compounds were detected better in ESI- mode, however, ESI+ spectra aided the identification of some compounds that lacked ESI- reference spectra or had more than one match. Figure 1 represents a DAD chromatogram of a *HII* MWE with compound numbers assigned to the peaks. The chromatogram was recorded at 280 nm, at which most of the phenolic compounds were detected.

The applied method of analysis, along with the help of identification keys reported by other researchers, also enabled the distinguishing between isobaric compounds and identification of structural isomers. In the following sub-sections, the identification procedure of compounds is described in greater detail, with the emphasis on those more difficult to identify and compounds or isomers, which have not been previously reported in *H. italicum*.

#### 2.1.1. Hydroxycinnamic Acids (Chlorogenic Acid Derivatives)

The class of chlorogenic acids (CGAs) represents a large group of compounds, among which the most characteristic are caffeoylquinic acids—conjugates of tetrahydroxy-cyclohexane carboxylic acid (quinic acid) and 3,4-dihydroxycinnamic acid (caffeic acid). Quinic acid can form even di- or tri-esters (e.g., di- or tri-caffeoylquinic acids; CQAs) or esters with several other trans-hydroxycinnamic acids, commonly ferulic and p-coumaric acids, which are then named feruloylquinic acid (FQA) and p-coumaroylquinic acid (pCoQA), respectively. Furthermore, ester mixes like caffeoyl-feruloylquinic acid (CFQA) can be formed as well, contributing to the even bigger complexity of this class [10,11].

Caffeic acid (**21**) was identified by comparing fragments with reference spectra. Four caffeic acid derivatives, three at *m*/*z* = 341 (**9**, **14**, **16**) and one at *m*/*z* = 567 (**57**) were observed. Compounds **9**, **14** and **16**, produced the same fragmentation ions corresponding to hexose moiety loss (162 Da) but with slightly different abundances and were identified as caffeic acid hexosides. Namely, the same [M−H]^−^ at *m*/*z* = 341 is also generated by caffeoyl hexoses, where caffeic acid is connected with sugar moiety through ester bond instead of ether but in that case fragments characteristic for sugar moiety fragmentation are observed [12]. The fourth compound with fragment ion at *m*/*z* = 341 was semi-identified as a caffeic acid O-hexoside derivative (**57**). Glycosides eluted before its aglycones, which is in accordance with literature, in which several caffeic acid hexoside isomers have been identified before in tomato products by Vallverdú Queralt et al. [13]. Compound **57** and isomers of caffeic acid glycosides, have not been reported in *H. italicum* extracts before.

At least three positional isomers of monoacyl-CQAs are known to be present in *H. italicum* [14]. They all have identical molecular formula C_16_H_18_O_9_ with 354.0951 as the accurate theoretical mass. Pseudomolecular ion [M−H]^−^ at *m*/*z* = 353 appeared at several retention times, indicating several CQA isomers. Compound **7** that eluted first was tentatively identified as a 3-O-CQA isomer (neochlorogenic acid), based on fragment ion abundances and literature data on retention times [13,15,16,17]. The most abundant was compound **15** (t_R_ = 6.95 min) and was identified as 5-O-caffeoyl-quinic acid (5-CQA) (chlorogenic acid), which is also the most common CGA in nature [11]. Spectra matched well with those in the library but was also further confirmed with an authentic standard. Another peak matching chlorogenic acid, which was also observed in the standard solution, was present. The latter was less abundant but with a very similar fragmentation pattern, suggesting geometric isomerism. As cis-5-acyl isomers are reported to be appreciably more hydrophobic than their trans counterparts, the later eluting peak was tentatively identified as cis-5-O-CQA (**25**) [18]. Another compound (**17**) with [M−H]^−^ at *m*/*z* = 353 was detected but presented quite different fragment abundances than features **7**, **15** and **25** mentioned earlier. Fragmentation ions at *m*/*z* = 173 and *m*/*z* = 3 were highly abundant, which is characteristic for 4-O-CQA, as was ion at *m*/*z* = 191, which should not be so intense. Despite that we identified that peak as such, due to retention times that were in line with the literature [17,18]. It has been reported in the literature previously, that CGAs can readily transform to one another, especially during extraction procedures at elevated temperatures and in the presence of water. Chlorogenic acid (5-O-CQA) not only isomerizes to 3- and 4-O-CQA but also undergoes other transformations such as esterification and reactions with water (i.e., hydrolysis) [19]. However, it is also possible that geometrical isomerism was induced already in the plant by activators of plant defense and priming responses, considering a significant amount of the second peak [17]. To the best of our knowledge, this is the first time that geometrical isomer of chlorogenic acid is identified in *H. italicum*.

FQAs are a group of derivatives with pseudomolecular ion [M−H]^−^ at *m*/*z* = 367. The same molecular mass fits also for caffeoylquinic acid methyl esters, among which 5-O-caffeoyl-4-methylquinic acid has been reported previously in *H. italicum* extract [20]. Compound **31** was identified as 5-O-FQA, as the base peak ion was at *m*/*z* = 191 and not 161 or 179, which are characteristic to methyl esters [15,21]. One feruloylquinic acid was also tentatively identified by Pereira et al. [8]. Ferulic acid (**65**) with [M−H]^−^ at *m*/*z* = 193 was identified at later elution times which is in accordance with Pereira et al. [8]. Similar derivatives are CoQAs, which have a molecular ion at *m*/*z* = 337. As such was identified compound **26**, whereas compound **34** produced a base fragment ion at *m*/*z* = 191 and was therefore tentatively identified as 5-O-CoQA [15]. Free coumaric acid (**40**) was also detected close after the compound **34**. Similar retention times are also observed in the literature [13]. Compounds **19** and **24** were identified as coumaric acid hexosides (*m*/*z* = 325), with identifier ions at *m*/*z* = 163 and 119 indicating the presence of coumaric acid and typical loss of CO_2_ [M−H−44]^−^ [13]. A coumaric acid hexoside has been reported in *H. italicum* previously by Pereira et al. [8].

As previously reported, both di-CQAs and CQA-glycosides produce an isobaric pseudomolecular ion at *m*/*z* = 515. Unlike the diCQA, the CQA glycosides produce distinctive ions at *m*/*z* = 341 ([caffeoyl glucoside-H]^−^) or/and 323 ([caffeoyl glucoside-H-H_2_O]^−^). A glycoside can be formed through an ether bond at either C-3 or C-4 on the aromatic caffeoyl ring. During MS fragmentation, these molecules give rise to ions at *m*/*z* = 341 which predominates in both cases, however, a peak at *m*/*z* = 323 is characteristic for glucosyl attachment at C-3 [17]. Compound **4** produced fragment ions only at *m*/*z* = 341 and was therefore tentatively identified as 3-O-(4′-O-caffeoyl glucosyl) quinic acid. For the compound **6**, product ions at both *m*/*z* = 341 and 323 were observed but the latter was less abundant. The predominating fragment ion was at *m*/*z* = 191 but at higher collision energies, an ion at *m*/*z* = 161 was also observed. This feature was therefore tentatively identified as 5-O-(4′-O-caffeoyl glucosyl) quinic acid. Compound **12** was the most intense one, producing a base fragment at *m*/*z* = 323, which indicated the presence of an ether bond at the 3′ position. Based on the abundances of other fragments, its identity was predicted to be 5-O-(3′-O-caffeoyl glucosyl) quinic acid. Compound **12** has been reported before by de la Garza et al. [22], while isomers **4** and **6** are reported here for the first time. At later eluting times, di-CQAs are eluting and the isomers can be characterized based on identification keys and information published elsewhere [16,17,18,23]. Compounds **46**, **49**, **51** and **56** were tentatively identified as 3,4-diCQA, 3,5-diCQA, 1,5-diCQA and 4,5-diCQA, respectively. This identification is in accordance with the results for *H. italicum* methanolic extracts obtained by Gonçalves et al. [24] and by Zapesochnaya et al. [14] in terms of the number of isomers present and identified.

Structurally related but much less common, are esters formed by the reaction of quinic acid alkyl ester (quinate) with caffeic acid or of quinate with ferulic acid. They do appear in nature, although their isolation and identification are rather difficult. Methyl quinates might as well be the product of extraction with methanol and could frequently be found as artefacts in plant analysis [21]. Di-CQA methyl esters produce a pseudomolecular ion [M−H]^−^ at *m*/*z* = 529 but so do FCQAs and FQA-glycosides. The parent ion at *m*/*z* = 529 was observed for compound **63**, with a base fragment ion at *m*/*z* = 367, which is characteristic for deprotonated FQA and a fragment at *m*/*z* = 193 for ferulic acid. No ion at *m*/*z* = 337 ([feruloyl glucoside-H_2_O-H]^−^) was present, to indicate an ether linkage with hexose [10]. Also, FQA-glycosides have a similar polarity as other CQA glucosides and are therefore expected to elute earlier. Based on that information and the identification key from Clifford et al. [15], this feature was tentatively identified as 3-feruloyl-5-caffeoylquinic acid. Another compound (**67**) with [M−H]^-^ at *m*/*z* = 529, produced a secondary product ion at *m*/*z* = 173 and was tentatively identified as 4-feruloyl-5-caffeoylquinic acid. Retention times were in accordance with Baeza et al. [25]. Pereira et al. [8] detected three features with a pseudomolecular ion at *m*/*z* = 529 but were tentatively identified as di-CQA methyl esters. 3,5-Di-CQA-methyl ester has been reported and unambiguously identified in *H. italicum* previously by Mari et al. [20] but with later retention times. In addition, the fragmentation profile of the 3,5-di-CQA-methyl ester isomer reported by Jaiswal and Kuhnert [21], did not match with the one observed by us.

TriCQAs produce a molecular ion at *m*/*z* = 677. For compound **36**, the parent ion at *m*/*z* = 677 was detected along with its characteristic fragment ions at *m*/*z* = 515, 353 and 191. However, Clifford et al. [12] suggest that if a tentative tri-CQA isomer elutes before diCQA, it is too hydrophilic to be tri-CQA. Namely, under that molecular mass appear also compounds with either an additional caffeic acid residue (diCQA glycosides, C_31_H_34_O_17_) or an additional hexose residue (CQA diglycosides, C_28_H_38_O_19_). Discrimination between the two was possible based on the slight difference in monoisotopic mass and compound **36** was therefore identified as di-CQA glycoside. Compound **72** with [M−H]^−^ at *m*/*z* = 677 and later retention time was identified as putative tri-CQA. Compounds with a pseudomolecular ion at *m*/*z* = 677 are reported in this study for the first time for *H. italicum*.

The unusual CQA derivatives were identified as well. Compound **52** was identified as tricaffeoylhexaric acid, based on the molecular ion at *m*/*z* = 695 and characteristic product ions at *m*/*z* = 533, 371 and 209. The proposed identification is in accordance with the literature, as this rare derivatives have been reported before for Asteraceae [26,27]. Similarly, compound **22**, with a molecular ion at *m*/*z* = 533, was putatively identified as dicaffeoylhexaric acid. Compound **59** was identified as another CQA derivative with [M−H]^−^ at *m*/*z* = 601 and with the main product ions at *m*/*z* = 395, 233 and 173. It has already been identified by Pereira et al. [8] as methoxyoxalyl dicaffeoylquinic acid. Compound **38**, with a pseudomolecular ion at *m*/*z* = 747 and fragment ions with *m*/*z* = 585, 422 and 459, was detected by Pereira et al. [8] as well but remained unidentified. The fragment at *m*/*z* = 585 was produced after a glucose/caffeoyl moiety loss (162 Da) but our attempt in its identification was just as unsuccessful. Namely, the only relevant report with that molecular ion was for isobutyryl diCQA, published by Kłeczek et al. [27], which together with 162 Da, does produce the observed molecular ion. However, the molecular formula corresponding to either isobutyryl diCQA glycoside (C_29_H_30_O_13_) or isobutyryl triCQA (C_34_H_30_O_15_) does not match the observed most probable molecular formula, which was calculated as C_34_H_36_O_19_. The compound **38** was therefore semi-identified just as a caffeoyl derivative.

#### 2.1.2. Hydroxybenzoic Acids and Their Glucosides

The first eluted compound (**1**) was identified as gallic acid glucoside, based on typical fragment ions corresponding to a hexoside group loss [M−H-162]^−^ and for CO_2_ loss [M−H-162-44]^−^. Compound **3** was identified as 3,4-dihydroxybenzoic (protocatechuic) acid O-hexoside with a pseudomolecular ion [M−H]^−^ at *m*/*z* = 315. It produced daughter ions at *m*/*z* = 153, corresponding to protocatechuic acid after the neutral loss of the hexoside group and at *m*/*z* = 109 produced after the neutral loss of a hexose moiety, followed by the neutral loss of CO_2_ (44 Da). This is a known phenolic compound [13,16] but has not yet been reported in *H. italicum*. However, its aglycone has been previously reported by Pereira et al. [8] and Gonçalves et al. [24] but the latter were unable to quantify it. Another compound (**23**) with a similar fragmentation pattern has been detected and its identification was predicted to be 2,4-dihydroxybenzoic acid. Compound **35**, with parent ion at *m*/*z* = 197, was semi-identified as a dihydroxybenzoic acid derivative. Compound **18** was identified as vanillic acid hexoside, due to molecular ion at *m*/*z* = 329 and the most intense fragment ion at *m*/*z* = 167. Compounds **8**, **27** and **29** with the same molecular ion at *m*/*z* = 329 and a somewhat improper fragmentation profiles were semi-identified as vanillic acid derivatives. Vanillic acid derivatives are common in plants but they have not yet been reported in *H. italicum*. Compounds **11** and **62** were identified based on their fragmentation profile and retention times as 4-hydroxybenzoic and 2-hydroxybenzoic acid (salicylic acid), respectively. Compound **13**, with a pseudomolecular ion at *m*/*z* = 299 and fragment ions at *m*/*z* = 137 and 93, corresponding to a hexose moiety loss and additional neutral loss of CO_2_, was identified as hydroxybenzoic acid hexoside. Compound **10** (*m*/*z* = 331), with fragments characteristic for hydroxybenzoic acid, was semi-identified as a hydroxybenzoic acid derivative. Compounds **10** and **13** are reported for *H. italicum* here for the first time.

#### 2.1.3. Flavonoids and Their Glycosides

The first eluting compound (**30**) of the flavonol class, presented a molecular ion at *m*/*z* = 609 and was tentatively identified as flavonoid dihexoside, due to the presence of a base peak at *m*/*z* = 285 [M−H-162-162]^−^ and at *m*/*z* = 447 [M−H-162]^−^. The aglycone was identified as kaempferol, based on characteristic fragment ions at *m*/*z* = 225 and 227. Compound **54** was identified as kaempferol glycoside (*m*/*z* = 447). Both have already been reported in *H. italicum* before [22,28,29]. Compound **61**, with a molecular ion at *m*/*z* = 489 and a fragment ion [M−H-204]^−^ at *m*/*z* = 285 produced after acetylhexoside loss, was therefore identified as kaempferol acetylglycoside, which is reported here for the first time. Compound **75** was detected with [M−H]^−^ at *m*/*z* = 593 or [M + H]^+^ at *m*/*z* = 595 and identified based on the fragmentation data reported previously [8] as tiliroside. Compounds **84** and **87** were identified as isokaempferides, presented by the matching fragment ions with those reported elsewhere [30]. Compound **81** was detected as kaempferol based on its characteristic fragment ions and compound **76**, with the same molecular ion at *m*/*z* = 285, was identified as its isomer luteolin, whose identity was also confirmed by reference standard comparison.

Compounds **33**, **41**, **45** and **64** all presented a molecular ion at *m*/*z* = 463 and a fragment ion at *m*/*z* = 301 and were identified as quercetin O-hexosides, which is in accordance with Pereira et al. [8], who also observed four features at *m*/*z* = 463. Quercetin 3-O-galactoside and 3-O-glucoside have been reported previously for *H. italicum* [31], whereas additional isomers have not been identified yet. Separation of these two 3-O-isomers is difficult and as a result, inconsistent data on their identification is reported in the literature [8,16,31]. Compounds **43** and **50** were identified as quercetin malonylhexosides ([M−H]^−^ at *m*/*z* = 549), due to fragment ions corresponding to the loss of acetylhexose (204 Da) after CO_2_ moiety loss (44 Da). Compounds **48** and **58** with a molecular ion at *m*/*z* = 477 produced fragments at *m*/*z* = 315 and 300, indicating a hexose moiety loss, followed by a methyl loss and were tentatively identified as isorhamnetin hexosides. Compound **58** produced slightly different fragments, which was in accordance with Pereira et al. [8]. Based on the information provided by Gu et al. [32], this derivative was probably isorhamnetin 3-O-glucoside. Compounds **77** and **83** were identified as isorhamnetin isomers. Similarly, compound **85** was identified as quercetin dimethyl ether, which has not been reported in *H. italicum* before. Compounds **68** and **70**, with a molecular ion at *m*/*z* = 609, were identified as quercetin coumaroylglucoside, as by Pereira et al. [8]. This conclusion was based on the fragment ions [M−H-146]^−^ at *m*/*z* = 463 and [M−H-162]^−^ at *m*/*z* = 301, which indicates the substitution of coumaric acid and hexose. Compound **28** was identified as quercetin diglycoside, analogous to the kaempferol derivative (**30**), which has also not been reported before. Compound **73** was identified as quercetin by comparing the fragmentation pattern with literature [13,16].

Compounds **32**, **39** and **47** were identified as myricetin derivatives. Compound **32** was identified as myricetin glucoside, which has already been reported for *H. italicum* [22], based on the fragment ion at *m*/*z* = 317, indicating a hexoside moiety loss and fragment ions characteristic for myricetin. Compound **39** with a parent ion at *m*/*z* = 565 and with the fragment ions corresponding to the loss of acetylhexose (204 Da) after a CO_2_ moiety loss (44 Da) was tentatively identified as myricetin malonylhexoside. This feature has been reported by Pereira et al. [8] but not identified. Similarly, compound **47** was identified as myricetin acetylglycoside but is reported here for the first time.

Compounds **90**, **91** and **37** were identified as methyl derivatives of known flavonols, based on literature data on their fragmentation patterns [8,33]. The first was tentatively identified as gnaphaliin, the second as galangin methyl ether and the last as herbacetin methyl ether. All three compounds were reported in *H. italicum* before [34]. Compound **55** was tentatively identified as herbacetin, based on its fragmentation profile.

Flavanones were much less abundant as above described flavonols. Compound **60** was identified as eriodictyol O-hexoside based on the molecular ion at *m*/*z* = 449 and a product ion corresponding to the loss of a hexoside moiety [M−H-162]^−^. Compound **71** was identified as its aglycone. Eriodictyol, as well as its glycoside derivative have not been reported in *H. italicum* previously but are common in other plant derived products [13]. Compounds **78** and **80** were identified as naringenin and its isomer. The first presented a good literature fragment match, whereas for the second, different fragment ions were more abundant. Compound **89** was identified as pinocembrin and also confirmed with an authentic standard, whereas compound **82** was identified as its isomer, due to matching spectra with pinocembrin as well. Compound **74** produced a fragment ion at *m*/*z* = 255 and was semi-identified as a pinocembrin derivative. The pinocembrin isomer and pinocembrin derivative have not been reported before.

#### 2.1.4. Coumarins

Compound **20** was identified based on the fragmentation profile as esculetin, which was also the most abundant coumarin present in the analyzed samples. It has also previously been identified in *H. italicum* extracts [35] but not in hydroalcoholic or water ones. The second detected coumarin was compound **42**, which was identified as scopoletin based on MS/MS spectra and retention time comparison with an authentic standard. In many samples, fragmentation did not occur, probably due to low concentrations.

#### 2.1.5. Arzanol and Other Pyrone Derivatives

Compound **93** was the most abundant compound of all the identified compounds. Its identity was confirmed as arzanol by comparison with a commercial reference standard. Arzanol is chemically characterized as prenylated heterodimeric phloroglucinyl α-pyrone. Compound **98** produced a very similar fragmentation profile to arzanol, so it was identified as an arzanol isomer. Compound **97**, with [M−H]^−^ at *m*/*z* = 567 and a fragment ion at *m*/*z* = 401, lead to its semi-identification as arzanol derivative. The arzanol isomer (**98**) and arzanol derivative (**97**) have not been reported before. Compound **96** appeared as a co-eluting peak and was tentatively identified as methylarzanol, based on the fragment ions comparison with Pereira et al. [8]. Compounds **92** and **99** were identified as heliarzanol 1 and 2, due to molecular ion at *m*/*z* = 445 and fragment ions similar to arzanol. However, no reference spectrum was available to confirm our findings. Compounds **95** and **100** were tentatively identified as italipyrone 1 and 2, also without the reference spectra to support it. The same counted for compound **86,** whose identity was proposed as micropyrone. Despite the absence of reference spectra, the identification is likely to be correct as these compounds were isolated from *H. italicum* before. For compound **94** reference spectra were available and it was identified as helipyrone, as was already reported previously [8,36].

#### 2.1.6. Other Phenolic Compounds

Compounds **44** and **53** were recognized as dihydrodehyrodiconiferyl glucoside derivatives, from the class of neolignans. The observed fragmentation pattern was plausible for the given formula. The observed fragments ions at *m*/*z* = 359 and 329 corresponded to the glucose moiety (162 Da) and sinapyl alcohol moiety loss (192 Da) followed by the loss of hydroxypropenyl moiety (46 Da). From the class of acetophenones, compound **88** was tentatively identified as 3-prenyl 4-hydroxyacetophenone, based on its fragmentation profile and reports from literature [8]. Compounds **66** and **69** were identified as 4-hydroxy-3-(2-hydroxy-3-isopentenyl) acetophenone 1 and 2, although reference spectra were not available. Isobenzofuranones and tremetones are the two groups of phenolic compounds that are readily extracted with methanol and have been previously reported in *H. italicum* [37]. However, they are rarely investigated in LC-MS studies and consequently reference spectral information is rather scarce. The reason for that lies in their lipophilicity, making them more suitable for GC-MS analysis [20]. Features **2** and **5** presented a molecular ion at *m*/*z* = 327 and product ions at *m*/*z* = 165 and 147, characteristic for a hexose moiety loss (162 Da) and additional water loss (18 Da). Based on the study by Lin et al. [38] and previous reports in *H. italicum* [37], they were identified as hydroxyphthalide glucosides. Compound **79** was identified as gnaphaliol glucopyranoside, a tremetone representative, based on fragment ions supporting the hexose moiety loss. No reference spectra were available.

### 2.2. Semi-Quantitative and Quantitative Analysis

**Figure 2:**
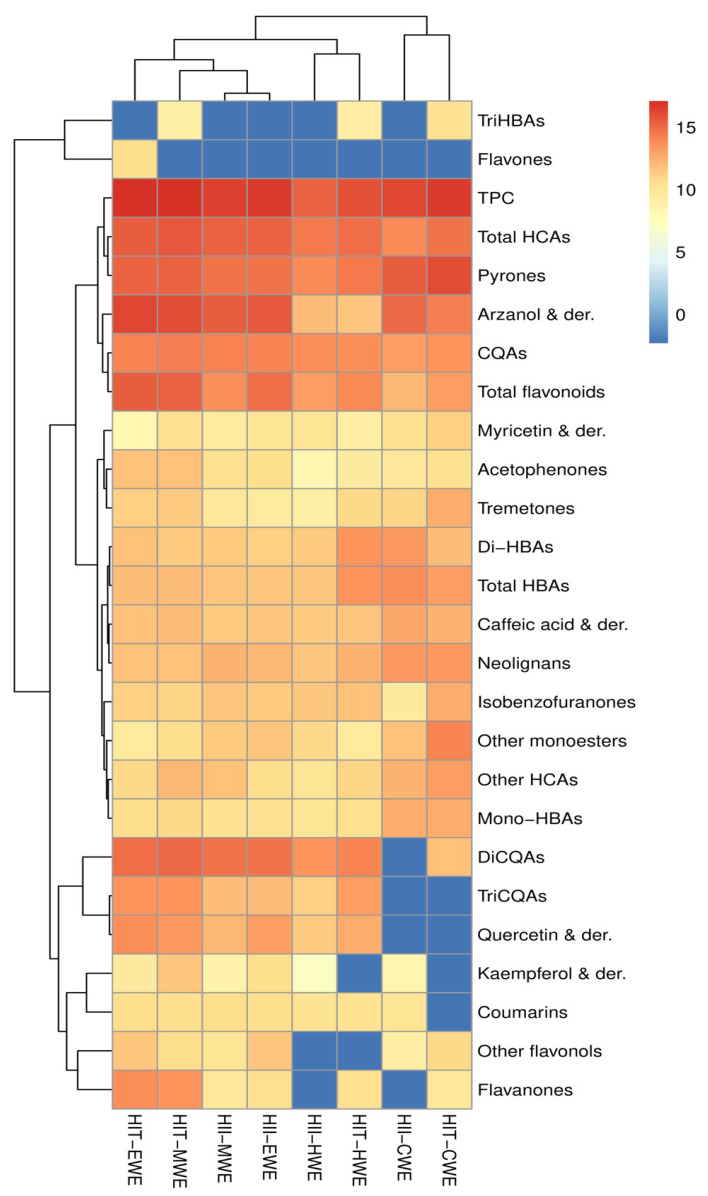
Heatmap representing the results of semi-quantitative analysis of putatively identified compounds in *H. italicum* extracts. Calculations were performed based on areas of extracted ion chromatograms (EICs), corrected for dilution factor during sample preparation. The abscissa is used to display the names of samples and the ordinate on the right is used to display the names of metabolites. The deeper the red color, the higher the content of the metabolites; the deeper the blue color, the lower the content of the metabolites. CQA—caffeoylquinic acids, HCA—hydroxycinnamic acids, HBA—hydroxybenzoic acids, TPC—total phenolic content (sum of all the identified and quantified compounds), der.—derivatives.

Some differences in DAD profiles arising from genetic diversity of *H. italicum* as well as extraction solvent used can be observed immediately. To better estimate which has greater impact on the chemical composition, semi-quantitative comparison of different *H. italicum* extracts was made by comparing areas of EICs of individually identified compounds as well as summed areas of compounds belonging to the same chemical class. The results are graphically presented in Figure 2, whereas areas for each identified compound can be found in Supplementary File, Table S1.

From the Figure 2 and the DAD chromatograms (Supplementary File, Figure S2), it can be seen that the EWEs and MWEs of the same species are the most alike, next to them are grouped the HWEs of both *HII* and *HIT* species, whereas CWEs are grouped completely separately. It is evident that the between-sample differences are primarily resulting from the extract preparation procedure, rather than the genetic differences of the subspecies. The differences in the composition of the analyzed extracts are understandable as the several extraction parameters, which can significantly influence the extraction yields of phenolic compounds, have been modified: extraction solvents (methanol, ethanol and water), temperature (room temperature and 100 °C), ultrasonic assistance and extraction time (5 days, 90 and 15 min). Preparation of extracts with water led to the loss of certain compound groups, such as di- and triCQAs, coumarins, quercetin and kaempferol derivatives in the case of cold water extraction with ultrasonic assistance, whereas other flavonols and flavanones were negatively affected in hot water extraction. To the contrary, some monoesters, diBAs, neolignans and caffeic acid derivatives, were more abundant in CWEs. On the other hand, some compound classes were basically unaffected by the extraction method used. Those were HCAs and pyrones, among which chlorogenic acid and arzanol were chosen for proper quantification.

The concentrations of two characteristic compounds in *H. italicum* extracts, obtained from calibration curves of the reference standards and the total phenolic content—TPC values are presented in Table 1. The TPC was calculated as a summation of all integrated DAD peaks and used for the comparison of the extracts’ strength.

Chlorogenic acid was the most abundant in *HIT* MWE and HWE, whereas the lowest was in CWE. Arzanol, on the other hand, was the most abundant in the *HII* EWE, whereas in both types of water extracts it was below the detection limit. However, with LC-ESI-QTOF-MS, arzanol was detected in all samples. It is not uncommon, to miss some phenolic compounds with DAD due to its lesser sensitivity. Nevertheless, it can be seen that the cold-water extraction, even with the ultrasonic assistance, gives the lowest yields for both compounds and that the ethanol:water extraction gave better yields of arzanol for both subspecies, whereas for chlorogenic acid this was true only for *HII*. The results for the TPC ranged from 19 ± 5 to 153 ± 1 mg CAE/g dry mass and were contrary to what was expected. The sample with the highest values was CWE of *HIT* and the HWE of *HII* with the lowest. The reason for that is most probably due to the ultrasonic extraction assistance, which accelerates diffusion and enhances the mass transfer phenomena and enables good extraction yields even at lower processing temperatures [19]. On the other hand, with hot water extraction, the boiling water increases the extraction but can in turn cause the breakdown of heat-sensitive compounds. With ultrasonic extraction, heat is also a negative factor but if the extraction time is reasonably short and heat production minimized with addition of ice to the water bath, this problem can be avoided. Significant contribution to TPC in case of CWE must be attributed to some unidentified phenolic compounds. Substituting methanol for ethanol had little but significant overall effect on TPC in case of *HII* and no impact in case of *HIT*. Furthermore, the reduction of methanol to water ratio (from 3:1 to 1:1) decreased the TPC significantly (data not shown) but did not have proportionally negative impact on the bioactivity of the extracts. Namely, during the method optimization, higher methanol concentration was used for the extraction but later on proved to be of minor importance for the bioactivity, therefore it was not tested further. Based on the present results, we can conclude that *HIT* plant is richer in overall phenolic compounds than *HII*.

### 2.3. Antioxidant Activity

From the antioxidant test with DPPH radical, several values indicating the antioxidant potential of the extracts were determined—the EC_50_ value, indicating the concentration of the extract needed to inhibit 50% of the DPPH radical; maximal inhibition (inh_max_), representing the highest value of DPPH inhibition; and the expression of the one tested concentration of the sample in mg of ascorbic acid equivalents per g of dried *H. italicum* material. The results are presented in Table 2.

The lowest EC_50_ value was determined for the *HIT* MWE and was only 4-fold bigger as for ascorbic acid, followed by EWE and then by the HWE. CWEs had a 6-fold weaker potential than HWEs. A similar trend was observed for *HII* extracts. The maximum inhibition observed in the tested concentration range was around 82% for most samples. In the case of CWEs, it was 86 or 75% for *HIT* and *HII* extracts, respectively. Contrary to the highest TPC, *HIT* CWE did not have the best antioxidant potential, which suggests that bioactive compounds were deactivated during the extraction process or that mostly weakly active polyphenols were extracted. On the other hand, HWEs had a higher antioxidant potential as expected from the TPC, especially in the case of *HII*. Results were also expressed as mg AAE per g of dry mass for easier comparison of the antioxidant potential, where the higher values indicate higher antioxidant potential.

**Figure 3:**
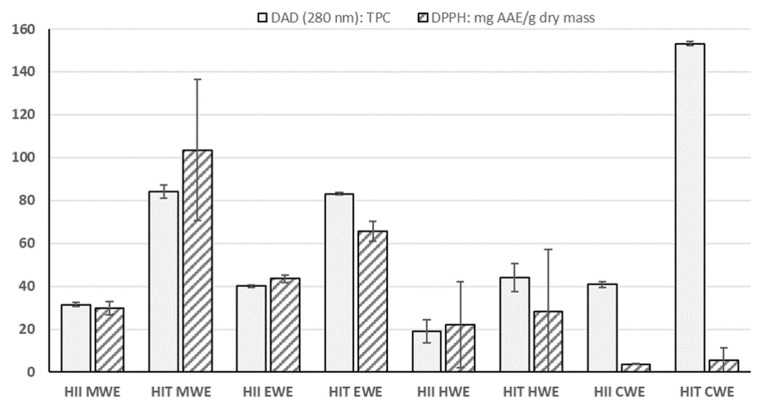
Representation of DPPH results in comparison with TPC. MWE—methanol:water extract, EWE—ethanol:water extract, HWE—hot water extract and CWE—cold water extract.

The relations between the antioxidant assay and TPC is shown in Figure 3. It can be seen that values correlated nicely for all the samples, except for CWEs. Pearson’s correlation coefficient of r = 0.89 (*p* < 0.001) was calculated when excluding CWEs. If the CWEs were included in the calculation, no significant relationships existed between TPC and antioxidant potential. This data further supports the findings, that cold water is not a good choice for extraction of the antioxidant active compounds, even with the assistance of ultrasound. Furthermore, the TPC and antioxidant activity were inversely proportioned for *HIT* (with the exception of methanol extract), whereas for *HII* antioxidant activity was higher compared to TPC values.

## 3. Materials and Methods

### 3.1. Reagents and Chemicals

Reference standards of chlorogenic acid, scopoletin, pinocembrin, arzanol and luteolin were purchased from Sigma-Aldrich (Merck KGaA, Darmstadt, Germany) and were of analytical or pharmaceutical primary standard grade. Mass spectrometer reference calibration mixtures and the tuning mix were purchased from Agilent (Agilent Technologies, Inc., Santa Clara, CA, USA). The mobile phase solvents methanol, water and formic acid were of LC-MS grade, whereas ethanol and methanol for extract preparation were of HPLC grade. All were purchased from Honeywell (Honeywell International Inc., Charlotte, NC, USA). Water used for cold and hot extraction was ultra-pure and obtained with the Purelab^®^ Option-Q water purification system (Evoqua Water Technologies LLC, Pittsburgh, PA, USA). DPPH and ascorbic acid were purchased from Sigma-Aldrich as well.

### 3.2. Plant Collection

For research proposes, the Ex Situ experimental collection of *Helichrysum italicum* (Roth) G. Don of the University of Primorska was established in 2018 near Ankaran (45°34′19.3″ N 13°46′33.2″ E), Slovenia. Plant material for the collection was obtained with inventarization of some private, Slovenian home gardens where different *H. italicum* phenotypes are maintained for a decade. Plant material for the preparation of seedlings was acquired from morphological different mother plants grown in home gardens and was vegetative propagated in the nursery. Two-year seedlings were planted in rows at a distance of 0.7 m × 0.4 m according to the randomized block design in the experimental Ex Situ field in a well-drained soil with a sandy-loam texture. Both subspecies in the collection thrive under the uniform growing and ecological conditions of the sub Mediterranean climate. All plants in the collection were morphologically evaluated with the revised taxonomic identification key for *H. italicum*, which was recently developed by Herrando-Moraira et al. [1]. Based on the qualitative and quantitative morphological characters of vegetative parts (presence of axillary leaf fascicles, caulinar leaf length, leaf margin) and floral part (number of capitula per synflorescence), two different morphological variants were identified. One variant of *H. italicum* was identified as *H. italicum* ssp. *italicum* (*HII*) and another as *H. italicum* ssp. *tyrrhenicum* (*HIT*). Plants in the collection thrive under the uniform growing and ecological conditions of the sub Mediterranean climate. Aerial parts of two morphological variants were harvested at the stage of development of generative shoots before their full flowering period in June 2019. Sampling of each variant was performed on five randomly selected plants. Stems and leaves, along with flower-tops were cut into smaller pieces, frozen with liquid nitrogen and freeze-dried (Alpha 1–4 LSCplus; Martin Christ Gefriertrocknungsanlagen GmbH, Osterode am Harz, Germany). Dried plant material was then stored at −20 °C until use.

### 3.3. Extraction Procedures

Essentially, two conventional extraction procedures were used for the preparation of the extracts: maceration and infusion. In the preliminary experiments different solvent ratios were tested as reported in previously published studies on *H. italicum* [22,39]. Hydroalcoholic extracts were prepared by maceration of the dried and milled plant material (2.5 g) of each *HII* and *HIT* with 50 mL of methanol:water (1:1) and ethanol:water (1:1) solvent mixtures, which were chosen as the most appropriate in terms of extraction yields and desired future applications. After 5-day maceration at room temperature in the dark, extracts were filtered through Whatman No. 41 filter paper and concentrated by a rotary evaporator (Rotavapor^®^ R-300; BÜCHI Labortechnik AG, Flawil, Switzerland). The dried residue was then dissolved in 5 mL of the original solvent mixture and kept at −20 °C until analysis. Cold water extracts were prepared by two successive macerations of 2.5 g of the plant material: first with 30 mL and the second with 20 mL of cold water, assisted with sonication (Elmasonic S 30 H, Elma Schmidbauer GmbH, Singen, Germany) in an ice bath for 60 min and 30 min, respectively. Ultrasonic assistance was used only in the case of cold water maceration, to improve poor extraction yields and shorten the time of maceration. Hot water extracts (infusions) were prepared just before analysis by immersing 1.25 g of milled plant material in hot water (50 mL) for 15 min and then filtered through filter paper. The drug to extract ratio used was the same as reported by Kazazic et al. [40]. Each extraction procedure was carried out in duplicates.

Samples for chemical analysis were diluted according to theirs estimated yields as follows: 1/50, 1/100 or 1/10 for hydroalcoholic, cold and hot water extracts, respectively, with the same solvent mixture as used for extraction. The diluted samples were passed through a 0.2 µm HPLC certified nylon membrane filter (Macherey-Nagel GmbH & Co KG, Düren, Germany) and kept in the amber HPLC vials (Agilent Technologies, Inc., Santa Clara, CA, USA) with PTFE/silicone septa caps at 4 °C until analysis.

### 3.4. HPLC-DAD-ESI-QTOF-MS Analysis

High performance liquid chromatography-mass spectrometry analysis of the reference standards and extracts samples was performed using an Agilent 1260 Infinity II HPLC system (Agilent Technologies, Santa Clara, CA, USA) equipped with a diode array detector (DAD, model G7115A) and coupled to an Agilent 6530 Accurate-Mass Quadrupole Time-of-Flight (Q-TOF) MS system equipped with an Agilent Jet Stream dual electrospray ionization (ESI) source. The HPLC system included a binary pump (model G7112B), Agilent 1260 Autosampler (model G7129A) and a Poroshell 120, EC-C18, 2.1 × 150 mm, 2.7 µm column (693775-902, Agilent Technologies, Santa Clara, CA, USA). The following method for the HPLC-MS analysis rests on our previous studies of phenolic compounds investigation [41,42]. Separation was obtained with a linear gradient of (A) water + 0.1% formic acid (*v*/*v*) and (B) acetonitrile/methanol (50:50, *v*/*v*), starting at 3.0 % B and increased to 100.0% B in 15 min and held for 5 min (flow rate 0.30 mL/min, column temperature 50 °C, injection volume 1 µL). The separated compounds were first monitored using DAD at 280 nm and 330 nm and then MS scans were performed under the following conditions: gas temperature 250 °C, drying gas flow 8 L/min, nebulizer 35 psig, sheath gas temperature 375 °C, sheath gas flow 11 L/min, capillary voltage 1000 V and fragmentor voltage 150 V. The ion-source parameters were the same in both positive and negative ESI modes. Mass spectra were recorded as centroid data for *m*/*z* 100–1000 in MS mode and *m*/*z* 40–1000 in MS/MS mode, with an acquisition rate of 14.0 spectra/sec. The Automated MS/MS data-dependent acquisition was done for ions detected in the full scan above 2000 counts with a cycle time of 0.5 s, a quadrupole isolation width in narrow ~1.3 Da, using fixed collision energies of 10, 20 and 40 eV and a maximum of three selected precursor ions per cycle. The instrument was tuned in low mass range (1700 *m*/*z*) and in extended dynamic range (2 GHz) mode. In those conditions, the instrument is expected to provide experimental data with accuracy within ±3 ppm. The Agilent MassHunter Data Acquisition software was used to acquire data.

All the acquired data were first processed using MassHunter Qualitative Analysis Workflows (version B.08.00) and Qualitative Navigator (version B.08.00) software. The extracts were screened for the range of phenolic compounds previously reported in *H. italicum* and identified, based on the accurate mass of precursor ions with minimum 80 overall match scores and fragmentation profile obtained from METLIN Metabolite and Chemical Entity Database (The Scripps Research Institute, San Diego, CA, USA) or literature data, if available. Targets with no reference MS/MS data available, were evaluated just on MS level and processed further with *in-silico* fragment prediction software—Molecular Structure Correlator (MSC). For qualitative and semi-quantitative between-sample comparison, Agilent’s Mass Profinder (version B.08.00) was used for simultaneous targeted feature extraction.

Quantification was performed by an external calibration method using chromatograms measured with DAD at 280 nm. Standard solutions (10 µg/mL) were prepared from dimethyl sulfoxide or methanol stock solutions in LC-MS grade methanol. Chlorogenic acid and arzanol reference standards were used to construct the calibration graphs and to quantify the two most characteristic compounds, which were identified in *H. italicum* extracts. The calibration plots indicated good correlations between peak areas and commercial standard concentrations with regression coefficients higher than 0.996. The lowest calibration point included in the calibration curve was used to calculate the limit of quantifications (LOQs). The results are expressed as mg of a standard per g of the dried *H. italicum* sample. The total phenolic content of an extract was determined as a summation of areas for all integrated peaks with signal-to-noise ratio greater than 5:1 and expressed as mg of chlorogenic acid equivalents (CAE) per gram of dry mass of the plant material.

### 3.5. Antioxidant Assay

The antioxidant activity of *H. italicum* extracts was measured in terms of their radical-scavenging ability in the DPPH radical assay. The assay was performed as reported previously by Zegura et al. [43], with minor modifications. Briefly, reaction mixtures containing 7.8 to 2500 µg/mL of extracts and 0.1 mM DPPH in methanol were incubated at ambient temperature for 60 min in 96-well microtiter plates in the dark. The decrease in absorbance of the free radical DPPH was measured at 515 nm with a microplate reader Infinite F200 (Tecan Group Ltd., Zürich, Switzerland). Ascorbic acid was used as a positive control. The free radical scavenging activity was calculated as the percentage of DPPH radical that was scavenged, as follows:

(1) $$\%\;radical\;scavenger\;activity=(1-\frac{A_{sample+DPPH}\;-\;A_{sample}}{A_{blank+DPPH}\;-\;A_{blank}})\;\times 100$$

A_sample+DPPH_: absorbance in the presence of *H. italicum* extracts or ascorbic acid,

A_blank+DPPH_: absorbance of the control reaction (solvent without *H. italicum* extracts),

A_sample_: absorbance of the sample,

A_blank_: absorbance of methanol.

EC_50_ values were determined graphically from the curves. Two independent experiments with at least three replicates each were performed. Results were also expressed as mg of ascorbic acid equivalents (AAE) per grams of dry plant material.

### 3.6. Statistical Analysis

The results were expressed as mean values ± standard deviation. One-way analysis of variance (ANOVA) and independent sample *t*-test were used to compare the differences in an antioxidant activity determined by DPPH test between two subspecies (*HII* vs. *HIT*) and between different extraction procedures. Levene’s test was performed to verify if there was homogeneity of variances. Pearson’s correlation analysis was performed to evaluate the relationship between total phenolic content (TPC) and antioxidant activity determined by DPPH test. All statistical outcomes with *p* values less than 0.05 (*p* < 0.05) were recognized as statistically significant. Statistical analyses were performed with the help of computer software—Statistical package for the social sciences (SPSS) version 23.0 (IBM Inc., Chicago, IL, USA). In addition, heatmap of hierarchical cluster analysis was conducted to present the results of semi-quantitative analysis of putatively identified compounds in *H. italicum* extracts. Calculations were performed based on areas of extracted ion chromatograms (EICs), corrected for dilution factor during sample preparation. Heatmap was conducted by the heatmap (Version 1.0.12) package of R software (Version 3.5.0).

## 4. Conclusions

The aim of this study was to examine the phytochemical profile of two *Helichrysum italicum* subspecies (*HIT* and *HII*) prepared with different extraction procedures and to investigate the differences between the extracts in terms of bioactive compounds resulting in possible distinction of antioxidant activity. In total, one hundred compounds were identified. Among them are several isomers and derivatives reported here for the first time (e.g., vanillic acid derivatives, di- and tricaffeoylhexaric acid, CoQA, FCQAs, CQA glucoside isomers, triCQA, eriodictyol and its derivatives). The most abundant compounds were caffeoylquinic acids and pyrones. This study is also noteworthy as it compares two subspecies of *H. italicum* grown under the same environmental conditions, between which no drastic differences in terms of qualitative composition were observed. Great similarities can also be drawn with the study of *H. italicum* ssp. *picardii* by Pereira et al. [13]. Although the morphological differences between the subspecies were obvious, a more accurate classification would only be possible with the help of DNA markers. Conversely, differences in the response to the extraction procedure and the parameters applied in the extraction process were evident. In case of *HII*, ethanol:water extracts gave better TPC yields than methanol:water, whereas the opposite was true for *HIT*. All *HIT* extracts had higher TPC content compared to *HII*, while the antioxidant potential was not proportionally higher. From these results, we can conclude that the antioxidant compounds in *HII* are either more potent but present in lesser amounts or that some non-phenolic substances contribute to the antioxidant activity. To better understand the mechanism of action and to confirm the potential use of these species in disease prevention or treatment, additional in vivo antioxidant assays are required. A key observation was that the hot water extracts proved to be comparably active as alcoholic ones, confirming the high commercial potential of *Helichrysum italicum* preparations as herbal functional beverages in the health-foods category. This study provided important information for selecting the best extract for further studies on the bioactivity of *Helichrysum italicum*.

## Figures and Tables

**Table 1:** Quantification of two most characteristic compounds (mg/g dried material) and the total phenolic content (TPC) of *H. italicum* extracts determined by DAD (280 nm).

Sample	Chlorogenic Acid	Arzanol	TPC ^1^
*HII* MWE	4.6 ± 0.1	2.9 ± 0.1	31.4 ± 0.9
*HIT* MWE	5.7 ± 0.3	2.1 ± 0.2	84 ± 3
*HII* EWE	5.2 ± 0.2	6.72 ± 0.07	40.0 ± 0.7
*HIT* EWE	5.1 ± 0.1	3.50 ± 0.05	83.1 ± 0.5
*HII* HWE	4 ± 1	<LOD	19 ± 5
*HIT* HWE	5 ± 1	<LOD	43 ± 6
*HII* CWE	<2.1	<LOD	40 ± 1
*HIT* CWE	3 ± 1	<LOD	153 ± 1

^1^ Results are expressed as chlorogenic acid equivalents (CAE) per dry mass. MWE—methanol:water extract EWE—ethanol:water extract, HWE—hot water extract, CWE—cold water extract, LOD—limit of detection.

**Table 2:** Antioxidant potential of *H. italicum* samples determined by DPPH test and expressed as EC_50_ and ascorbic acid equivalents (AAE).

Sample	EC_50_ [µg/mL]	Inh_max_ [%]	mg AAE/g Dry Mass
Ascorbic acid	3.5	90.2	/
*HII* MWE	26 ± 1 ^a,b^	83 ± 3	30 ± 3 ^a^
*HIT* MWE	15 ± 1	82.75 ± 0.07	104 ± 33 ^c^
*HII* EWE	20 ± 1	83.0 ± 0.6	44 ± 2 ^a^
*HIT* EWE	19 ± 7	83 ± 2	66 ± 5
*HII* HWE	41 ± 4 ^a,d^	83.2 ± 0.9 ^d^	22 ± 20 ^d^
*HIT* HWE	29 ± 4 ^e^	82.8 ± 0.8 ^e^	28 ± 29 ^e^
*HII* CWE	268 ± 67 ^a^	75 ± 5 ^a^	3.8 ± 0.3 ^a^
*HIT* CWE	171 ± 63	86.6 ± 0.9	5.7 ± 0.9

MWE—methanol:water extract, EWE—ethanol:water extract, HWE—hot water extract and CWE—cold water extract. ^a^ Statistical significance (*p* < 0.01) between *HII* and *HIT* (MWE; EWE; CWE; HWE), ^b^ between *HII* MWE and *HII* EWE, ^c^ between *HIT* MWE and *HIT* EWE, ^d^ between *HII* HWE and *HII* CWE, ^e^ between *HIT* HWE and *HIT* CWE.

## Supplementary Materials

The following are available online at https://www.mdpi.com/2218-1989/10/10/403/s1, Figure S1: Total ion chromatograms of the tested samples gathered in negative (A) and positive (B) ESI mode. Samples are numbered accordingly: (a) *H. italicum* ssp. *italicum* (*HII*) methanol:water extract in ratio 3:1 and (b) *HII* methanol:water extract in equal ratios, (c) *H. italicum* ssp. *tyrrhenicum* (*HIT*) methanol:water extract in equal ratios, (d) and (e) *HII* and *HIT* ethanol:water extracts in equal ratios, (f) and (g) *HII* and *HIT* hot water extracts, (h) and (i) cold water extracts., Figure S2: DAD chromatograms at 280 nm of the *H. italicum* samples overlaid accordingly: (A) and (B) representing differences in chemical profile between methanol and ethanol extracts of *H. italicum* ssp. *italicum* (*HII*) and *H. italicum* ssp. *tyrrhenicum* (*HIT*), respectively (C) and (D) representing differences in ethanol versus hot water extracts of the *HII* and *HIT*, respectively, (E) and (F) representing differences between *HII* versus *HIT* methanol and hot water extracts, respectively, Table S1: Results of the semi-quantitative analysis for the detected compounds in all tested samples. Values were calculated based on areas of extracted ion chromatograms (EICs) and corrected for dilution factor during sample preparation.

## Appendix A

**Table A1:** Comprehensive chromatographic and spectral data of the (tentatively) identified compounds in *H. italicum* extracts obtained with HPLC-DAD-ESI-QTOF-MS.

No.	RT (Min)	Compound Name	Compound Formula	Molar Mass	Diff. (ppm)	*m*/*z* ESI−	MS2 ESI−	*m*/*z* ESI+	MS2 ESI+	UV_max_ (nm)	Level of Identification ^1^
1	2.90	Gallic acid glucoside	C13H16O10	332.0757	−3.35	331.0682	331.1; 168.0; 125.0; 167.0; 149.9; 124.0;				NPR/MS2
2	4.25	Hydroxyphthalide glucoside 1	C14H16O9	328.079	−0.24 0.92	327.0722 373.0773	165.1; 146.9; 103.0; 93.0; 77.0	329.0857	167.0; 121.0; 111.0; 149.0		PR in [37]/MS
3	4.84	Protocatechuic acid O−hexoside	C13H16O9	316.0789	1.83	315.0720	315.1; 152.0; 153.0; 108.0; 109.0			210	NPR/MS2
4	5.49	CQA−glucoside: 3−O−(4′−caffeoyl glucosyl) quinic acid	C22H28O14	516.1486	−1.24	515.1413	515.1; 179.0; 341.1; 173.0; 353.1; 135.0				NPR/MS2
5	5.55	Hydroxyphthalide glucoside 2	C14H16O9	328.079	1.17	327.0717	327.1; 165.0; 146.9; 121.0			207	PR in [37]/MS
6	5.95	CQA−glucoside: 5−O−(4′caffeoyl glucosyl) quinic acid	C22H28O14	516.1469	1.87	515.1400	515.1; 191.1; 353.1; 341.1; 179.0; 323.1; 135.0; 161.0	517.1547	163.0; 325.1; 135.0	220; 324	NPR/MS2
7	6.00	Caffeoylquinic acid: 3−O−CQA *(neochlorogenic acid)*	C16H18O9	354.0947	−0.11	353.0875	353.1; 191.1;179.0; 135.0; 146.9; 107.0	355.101	163.0; 145.0; 135.0; 117.0; 89.0		PR in [14]/MS2
8	6.08	Vanillic acid derivative 1	C14H18O9	330.0949	0.31	329.0876	329.1; 167.0; 123.0; 149.0; 102.9; 79.0			220; 204 280	NPR/SI
9	6.18	Caffeic acid hexoside 1	C15H18O9	342.0956	−1.76	341.0884	179.0; 341.1; 229.9; 135.0; 205.0; 133.0			202	NPR/MS2
10	6.28	Hydroxybenzoic acid derivative	C14H20O9	332.0543	−3.49	331.0471	165.0; 123.0; 121.0; 93.0; 77.0; 137.0; 79.0				NPR/SI
11	6.47	*p*−Hydroxybenzoic acid	C7H6O3	138.0313	0.19	137.0244	93.0; 137.0; 65.0; 41.0; 75.0; 49.0; 67.0			318; 226	PR in [24]/MS2
12	6.58	CQA−glucoside: 5−O−(3′−O−caffeoyl glucosyl) quinic acid	C22H28O14	516.1477	−0.31	515.1404	515.1; 323.1; 191.1; 341.1; 161.0; 85.0	517.1546	163.0; 325.1; 517.1; 145.0; 85.0; 135.0		PR in [22]/MS2
13	6.75	Hydroxybenzoic acid hexoside	C13H16O8	300.0843	0.54	299.0771	137.0; 299.1; 179.0; 59.0; 93.0; 71.0; 65.0			213	NPR/MS2
14	6.82	Caffeic acid hexoside 2	C15H18O19	342.095	−0.14	341.0874	341.1; 179.0; 135.0; 84.9; 73.9; 153.1			326; 297; 194; 218; 244	NPR/MS2
15	6.88	Caffeoylquinic acid: 5−O−CQA *(chlorogenic acid)*	C16H18O9	354.0953	0.49	353.0878	191.0; 353.1; 161.0; 85.0; 127.0; 87.0; 133.0; 83.0	355.1013	163.0; 135.0; 117.0; 135.0; 145.0		PR in [14,22,24]/ STD
16	7.01	Caffeic acid hexoside 3	C15H18O9	342.0951	−0.01	387.0932 341.0894	179.0; 341.0; 135.0; 107.0			216; 259; 288; 330	NPR/MS2
17	7.04	Caffeoylquinic acid: 4−O−CQA *(cryptochlorogenic acid)*	C16H18O9	354.0951	−0,07	353.0878 399.0944	191.1; 353.1; 173.0; 179.0; 161.0; 135.0; 93.0; 85.0	355.1011	163.0; 193.0; 145.0; 135.0; 117.0; 89.0; 193.0		PR in [14]/MS2
18	7.05	Vanillic acid O−hexoside	C14H18O9	330.0951	−0.16	329.0879	167.0; 329.1; 99.0; 230.7; 123.0; 125.0; 41.0				NPR/MS2
19	7.08	Coumaric acid hexoside 1	C15H18O8	326.1009	−2.2	325.0930	163.0; 325.1; 119.0;			214	PR in [8]/MS2
20	7.18	Esculetin	C9H6O4	178.0266	0.2	177.0193	177.0; 133.0; 89.0; 41.0; 79.0; 53.0;			326; 297; 194; 218; 240	PR in [35]/MS2
21	7.24	Caffeic acid	C9H8O4	180.0419	2.12	179.0346	135.0; 179.0; 164.0; 134.0; 45; 89.0				PR in [22,35]/MS2
22	7.26	Dicaffeoylhexaric acid	C23H34O14	534.1017	−0.94	533.0939	371.1; 533.1; 209.0; 191.0; 85.0; 57.0; 147.0; 179.0				NPR/MS2
23	7.45	2,4−Dihydroxybenzoic acid	C7H6O4	154.0259	4.21	153.0187	109.0; 153.0; 108.0; 65.0; 91.0			210; 245; 325; 295	NPR/MS2
24	7.51	Coumaric acid hexoside 2	C15H18O8	326.0997	1.1	325.0925	265.1; 163.0; 205.0; 161.0; 145.0; 119.0; 117.0; 59.0			325;298; 210; 245	PR in [8]/MS2
25	7.53	Caffeoylquinic acid: *cis*−5−O−CQA (*cis*−*chlorogenic acid*)	C16H18O9	354.095	0.18	353.0878	191.1; 353.1; 85.0; 111.0; 127.0; 135.0	355.1015	163.0; 254.9; 145.0; 111.0; 117.0; 135.0; 89.0		NPR/MS2
26	7.60	Coumaroylquinic acid	C16H18O8	338.1011	−3.31	337.094	191.1; 337.1; 87.0; 69.0; 163.0; 93.0; 43.0; 67.0;				NPR/MS2
27	7.71	Vanillic acid derivative 2	C14H18O9	330.0955	−0.53	329.088	167.0; 329.1; 108.0; 191.0			211; 204	NPR/SI
28	7.73	Quercetin diglycoside	C27H30O17	626.15	−1.41	625.1419	625.1; 593.1; 301.0; 463.1; 488.9; 300.0				NPR/MS2
29	7.80	Vanillic acid derivative 3	C14H18O9	330.0955	−1.89	329.0878	191.0; 167.0; 329.1; 123.0; 83.0; 81.0			216; 204	NPR/SI
30	7.97	Kaempferol diglycoside	C27H30O16	610.1535	−1.76	609.1472	609.2; 283.0; 49.4; 441.2; 285.0; 447.1; 328.0			200; 218; 180; 345	NPR/MS2
31	7.97	5−O−Feruloylquinic acid	C17H20O9	368.1094	−3.62	367.1037	191.1; 367.1; 173.0; 87.0; 93.0; 134.0; 85.0	369.1186	177.1; 369.1; 145.0; 117.0; 149.1		PR in [8]/MS2
32	8.0	Myricetin glycoside	C21H20O13	480.0914	−2.05	479.0842	479.1; 317.0; 316.0; 165.98; 139.0	481.0969	319.0; 481.1; 435.1; 169.0; 137.0		PR in [22]/MS2
33	8.15	Quercetin O−hexoside 1	C21H20O12	464.0959	−0.24	463.0883	463.1; 300.0; 301.0; 137; 300.0				PR in [20]/MS2
34	8.20	5−O−Cumaroylquinic acid	C16H18O8	338.1001	−0.57	337.0931	191.1; 337.1; 145.0; 163.0, 81.0; 85.0; 93.0			320; 295; 195; 234	NPR/MS2
35	8.3	Dihydroxybenzoic acid derivative	C9H10O5	198.0532	−2.67	197.455	197.0; 153.1; 138.0; 123.0; 109.0; 108.0; 137.0			320; 295; 195, 234	NPR/SI
36	8.37	Dicaffeoylquinic acid glycoside	C31H34O17	678.1806	−1.47	677.1734	677.2; 515.1; 353.1; 179.0; 191.1; 323.1				NPR/MS2
37	8.40	Herbacetin methyl ether	C16H12O17	316.0583	0.6	315.0508	315.0; 287.1; 255.0; 283.0; 227.0; 211.0; 183.0			216; 257; 291	PR in [34]/MS2
38	8.42	Caffeoyl derivative	C34H36O19	748.1856	−1.71	747.1791	747.2; 585.1; 422.1; 459.1				PR in [8]/SI
39	8.49	Myricetin malonylhexoside	C24H22O16	520.0861	−1.39	565.0843	521.1; 317.0; 565.1; 178.9; 174.0; 161.0				NPR/MS2
40	8.56	Coumaric acid	C9H8O3	164.0472	1.06	163.0399	119.0; 163.0; 91.1; 93.0; 117.0;			203; 228; 284; 340	PR in [44,45]/MS2
41	8.59	Quercetin O−hexoside 2	C21H20O12	464.0955	−3.28	463.097	463.1; 301.0; 300.0; 271	465.103	303.0; 465.1; 392.1; 229.0; 153.0; 285.0		PR in [31]/MS2
42	8.61	Scopoletin	C10H8O4	192.0431	−3.39	191.0359	176.0; 191.0; 148.0; 59; 104.0; 102.9; 120.0				PR in [35]/STD
43	8.64	Quercetin malonylhexoside 1	C24H22O15	550.0973	−2.05	549.0897	505.1; 301.0; 445.1; 300.0				NPR/MS2
44	8.66	Dihydrodehyrodiconiferyl glycoside derivative 1	C27H36O13	568.2157	−0.21	567.2085	567.2; 359.1; 341.1; 521.2; 329.1; 44.9;				NPR/MS2
45	8.70	Quercetin O−hexoside 3	C21H20O12	464.0958	−0.29	463.0882	463.1; 300.0; 301.0; 271.0; 255.0; 151.0	465.1027	303.0; 145.0; 85.0; 229.0; 97.0	196; 218: 244; 302; 328	PR in [31]/MS2
46	8.79	Dicaffeoyl quinic acid isomer: 3,4−diCQA	C25H24O12	516.1264	0.76	515.1189	515.1; 353.1; 203.0; 173.0; 179.0; 191.1; 135.0	517.1339	163.0; 499.1; 135.0; 145.0; 89.0; 117.0		PR in [14]/MS2
47	8.83	Myricetin acetylglycoside	C23H22O14	522.1012	0.28	521.0935	521.1; 503.1; 127; 367.1; 152.0; 108.0; 179.0				NPR/MS2
48	8.87	Isorhamnetin hexoside 1	C22H22O12	478.1121	−0.09	477.1039	477.1; 315.1; 314.0; 300.0; 299.0; 271.0; 201.0; 179.0			196; 218: 244; 302; 328	PR in [20]/MS2
49	8.90	Dicaffeoyl quinic acid isomer: 3,5−diCQA	C25H24O12	516.1268	0.01	515.1193	353.1; 515.1; 191.1; 179.0; 135.0; 173.0; 161.0;	517.1331	163.0; 499.1; 337.1; 145.0; 135.0; 117.0		PR in [14]/MS2
50	8.90	Quercetin malonylhexoside 2	C24H22O15	550.0963	−0.58	549.0931	505.1; 300.4; 301.0; 287; 271.0	551.1028	303.0; 551.1; 127.0; 85.0; 109.0		NPR/MS2
51	9.07	Dicaffeoyl quinic acid isomer: 1,5−diCQA	C25H24O12	516.1271	−0.14	515.1196	353.1; 515.1; 191.1; 179.0; 135.0	517.1332	163.0; 499.1; 319.1; 145.0; 89.0; 117.0	196; 218: 244; 302; 328	PR in [14]/MS2
52	9.11	Tricaffeoyl hexaric acid	C33H28O17	696.1331	−0.14	695.1255	695.1; 533.1; 371.1; 209.0; 85.0; 191.0				NPR/MS2
53	9.17	Dihydrodehyrodiconiferyl glycoside derivative 2	C27H36O13	568.2165	−1.4	567.2091	521.2; 491.2; 503.2; 476.2; 250.1; 329.1; 341.1; 99.0;			196; 218: 244; 302; 328	NPR/MS
54	9.20	Kaempferol glycoside	C21H20O11	448.101	−1.07	447.0936	447.1; 284.0; 285.0; 255.0; 227.0				PR in [22]/MS2
55	9.21	Herbacetin	C15H10O7	302.0428	−0.94	301.0357	301.0; 268.0; 133.0; 211.0; 135.0; 159.0; 132.0	303.0496	303.0; 313.0; 163.0; 137.1; 135.0; 123.0; 169.0; 285.0		NPR/MS2
56	9.23	Dicaffeoyl quinic acid isomer: 4,5−diCQA	C25H24O12	516.1268	−0.11	515.1192	515.1; 353.1; 173.0; 179.0; 191.1; 135.0; 93.0	517.1333	163.0; 499.1; 145.0; 135.0; 117.0		PR in [14]/MS2
57	9.29	Caffeic acid O−hexoside derivative	C27H36O13	568.2137	2.16	567.2071	179.1; 521.2; 341.1; 161.0; 326.1; 89.0; 71.0			207; 247, 305; 325	NPR/SI
58	9.33	Isorhamnetin hexoside 2	C22H22O12	478.1118	−3.25	477.1051	477.1; 315.0; 314.0; 243.0; 271.0; 285.0; 299.0	479.1177	317.1; 479.1; 85.0; 302.0; 153.0;		PR in [20]/MS2
59	9.35	Methoxyoxalyl dicaffeoylquinic acid	C28H26O15	602.128	−1.92	601.121	395.1; 439.1; 557.1; 353.1; 233.1; 191.0; 173.0; 179.0				PR in [8]/MS2
60	9.44	Eriodictyol O−hexoside	C21H22O11	450.1193	−3.96	449.1107	449.1; 287.1; 135.0; 151.0; 123.0; 152.0				NPR/MS2
61	9.47	Kaempferol acetylglycoside	C23H22O12	490.1121	−5.52	489.1065	489.1; 417.0; 285.0; 284.0; 255.0; 227.0				NPR/MS2
62	9.59	Salicylic acid	C7H6O3	138.0313	0.99	137.0244	93.0; 137.0; 65.0; 53.0			194; 224	PR in [8]/MS2
63	9.61	3−Feruloyl−5−caffeoylquinic acid	C26H26O12	530.143	−2.38	529.1364	367.1; 529.1; 193.0; 191.1; 134.0; 113	531.1493	177.1; 513.1; 531.2; 509.0; 163.0; 145.0; 117.0	295; 316; 250	NPR/MS2
64	9.66	Quercetin O−hexoside 4	C21H20O12	464.096	−1.07	463.0886	463.1; 301.0; 151.0; 178.9; 300.0; 121.0	465.1027	303.0; 465.1; 85.0; 97.0; 153.0; 61.0		PR in [31]/MS2
65	9.73	Ferulic acid	C10H10O4	194.0579	−0.21	193.0504	193.0; 161.0; 134.0; 132.0; 133.0; 104.0				PR in [8,45]/MS2
66	9.84	4−hydroxy−3−(2−hydroxy−3− isopentenyl) acetophenone 1	C13H16O3	220.1095	1.94	219.1022	219.1; 201.1; 119.0; 218.9; 45; 189.1; 157.1; 41.0			196	PR in [46]/MS
67	9.90	4−Feruloyl−5−caffeoylquinic acid	C26H26O12	530.1433	−3.1	529.1368	529.1;367.1; 353.1; 173.0; 193.1; 161.0				NPR/MS2
68	9.98	Quercetin coumaroylglucoside 1	C30H26O14	610.1321	−0.6	609.1254	609.1; 463.1; 300.0; 301.0; 255.0; 272.0;	611.1393	147.0; 309.1; 303.0;	208; 225; 265; 315; 360; 194; 292	PR in [8]/MS2
69	10.04	4−hydroxy−3−(2−hydroxy−3− isopentenyl) acetophenone 2	C13H16O3	220.1095	1.79	219.1022	219.1; 201.1; 119.0; 218.9; 45; 143.0; 185.1;				PR in [46]/MS
70	10.17	Quercetin coumaroylglucoside 2	C30H26O14	610.1319	−1.91	609.1253	609.1; 463.1; 301.0; 300.0; 151.0; 255.0;			204; 222; 295; 330	PR in [8]/MS2
71	10.24	Eriodictyol	C15H12O6	288.0641	−1.76	287.0566	151.0; 287.1; 135.0; 65.0; 68.0; 41.0				NPR/MS2
72	10.27	Tricaffeoylquinic acid	C34H30O15	678.1579	0.89	677.1502	677.2; 515.1; 631.1; 515.1; 353.1; 179.0; 173.0; 335.1; 161.0. 191.1				NPR/MS2
73	10.49	Quercetin	C15H10O7	302.0431	−0.59	301.0356	301.0; 179.0; 151.0; 121.0; 65.0; 63.0; 83.0; 107.0;93.0	303.049	−	192; 225	PR in [8]/MS2
74	10.49	Pinocembrin derivative	C27H32O15	596.1749	1.65	595.1678	549.2; 255.1; 279.1; 297.1				NPR/SI
75	10.50	Tiliroside	C30H26O13	594.1389	−2.71	593.131	593.1; 285.0; 284.0; 255.0; 145.0	595.1456	147.0; 309.1; 287.1; 165.1; 291.1; 91.1		PR in [8,20]/MS2
76	10.53	Luteolin	C15H10O6	286.0483	−0.46	285.0406	285.0; 151.0; 133.0; 107.0; 132.0; 63.0; 65.0;				PR in [22]/STD
77	10.91	Isorhamnetin 1	C16H12O7	316.0591	−2.45	315.0518	315.0;300.0; 271.0; 243.0; 255.0; 227.0			192; 225	PR in [8,34]/MS2
78	11.06	Naringenin	C15H12O5	272.0683	0.61	271.061	271.1; 151.0; 119.0; 65.0; 63.0; 41.0				PR in [22,47]/MS2
79	11.12	Gnaphaliol glucopyranoside	C19H24O9	396.1423	−0.7	395.135 441.1419	233.1; 395.1; 190.9; 146.9; 189.1; 83.0; 41.0; 191.1			195; 335	PR in [37]/MS
80	11.34	Naringenin isomer	C15H12O5	272.0683	0.61	271.061	271.1; 253.0; 151.0; 197.1; 63.0; 65.0; 83.0; 125.0;	273.0751	227.1; 153.0; 255.1; 273.1; 199.1;	209; 292	PR in [8]/MS2
81	11.37	Kaempferol	C15H10O6	286.0477	0.97	285.0405	285.0; 123.0; 185.1; 169.1; 159.0; 93.0; 155.1; 117.0				PR in [8,20,22]/ MS2
82	11.40	Pinocembrin isomer	C15H12O4	256.0741	−2.53	255.0669	255.1; 153.1; 213.0; 151.0; 171.0; 63.0; 65.0; 83.0				NPR/MS2
83	11.63	Isorhamnetin 2	C16H12O7	316.0596	−4.15	315.0525	315.0; 300.0; 60.7; 149.0; 271.0; 107.0; 83.0; 255.0				PR in [8,34]/MS2
84	11.78	Isokaempferide 1	C16H12O6	300.0632	0.69	299.0559	299.1; 284.0; 256.0; 255.0; 239.0; 227.0; 132.0; 183.0			205; 290	PR in [34]/MS2
85	11.85	Quercetin dimethyl ether	C17H14O7	330.0749	−2.53	329.0675	329.1; 314.0; 299.0; 271.0; 133.0; 215.0;	331.0807	331.1; 316.1; 301.0; 273.0; 217.0; 121.0		NPR/MS2
86	11.90	Micropyrone	C14H20O4	252.1345	0.01	251.1287	251.1; 207.1; 113.1; 151.1; 123.1; 85.1; 55.0				PR in [36]/MS
87	12.29	Isokaempferide 2	C16H12O6	300.0634	−0.06	299.0567	299.1; 284.0; 256.0; 255.0; 239.0; 227.0; 132.0; 183.0	301.0707	301.1; 107.1; 269.2; 286.0; 285.0; 212.0		PR in [34]/MS2
88	13.16	3−Prenyl−4− hydroxyacetophenone	C13H16O2	204.1147	−1.45	203.108	203.1; 148.1; 71.0; 105.0			197; 228; 290	PR in [46]/MS2
89	13.21	Pinocembrin	C15H12O4	256.074	−1.68	255.0667	255.1; 59.0; 151.0; 187.1; 213.0; 65.0; 83.0; 137.0				PR in [34]/STD
90	13.81	Gnaphaliin	C17H14O6	314.0797	−2.07	313.07	313.1; 298.0; 283.0; 227.0; 255.0; 199.0; 139.1; 183.0	315.086	315.1; 300.1; 285.0; 257.0; 138.9	210; 280	PR in [8,34]/MS2
91	13.87	Galangin methyl ether	C16H12O5	284.0689	−1.44	283.0615	283.1; 268.0; 211.0; 239.0; 167.1; 195.0	285.075	285.3; 270.0; 269.0; 136.0; 241.0; 168.1		PR in [34]/MS2
92	15.53	Heliarzanol 1	C24H30O8	446.1941	−0.8	445.1871	445.2; 279.1; 291.1; 235.1; 247.1; 205.1; 193.1; 191.1;			210; 295	PR in [44]/MS
93	15.65	Arzanol	C22H26O7	402.167	−1.95	401.1606	401.2; 235.1; 247.1; 153.1; 191.1; 109.1; 205.1; 166.0;	403.1743	249.1; 347.1; 237.1; 181.0; 403.2; 155.1; 193.0; 163.1;		PR in [8,36]/STD
94	16.21	Helipyrone	C17H20O6	320.1258	−1.09	319.1189	153.1; 109.1; 198.1; 41.0	321.1318	155.1; 321.1; 167.1; 139.1; 81.1; 57.0; 43.0	210; 230; 295	PR in [8,36]/MS2
95	16.57	Italipyrone 1	C22H24O7	400.1531	−0.66	399.1452	233.1; 399.1; 245.1; 153.1;109.1; 189.1	401.1601	401.2; 247.1; 235.1; 167.1; 187.0	19894	PR in [36]/MS
96	16.69	3−Methylarzanol	C23H28O7	416.1836	−0.23	415.1769	415.2; 261.1; 249.1; 205.1; 180.0; 153.1; 109.1; 193.1	417.1909	263.1; 251.1; 361.1; 195.1; 155.1; 207.1; 177.1; 189.1	199; 225; 275	PR in [8,48]/MS2
97	17.45	Arzanol derivative	C31H36O10	568.2318	−2.35	567.2249	567.2; 401.2; 413.2; 247.1; 235.1; 221.1; 191.1; 179.0			208; 295	NPR/SI
98	17.92	Arzanol isomer	C22H26O7	402.167	−1.95	401.1606	401.2;235.1; 153.1; 247.1; 109.1; 179.0; 166.0; 191.1			215; 295	NPR/MS2
99	17.97	Heliarzanol 2	C24H30O8	446.1956	−4.58	445.1888	445.1; 279.1; 291.1; 261.1; 109.1				PR in [44]/MS
100	18.73	Italipyrone 2	C22H24O7	400.1533	−3.82	399.1455	233.1; 399.1; 245.1; 153.1; 109.1; 189.1	401.1595	401.2;235.1; 247.1; 193.0	292; 214	PR in [36]/MS

^1^—different confidence levels of identification: PR—previously reported compound and the most relevant reference, NPR—not previously reported (new compound) in *H. italicum*, STD—confirmed with standard comparison, MS2—confirmed with fragment pattern comparison (METLIN or literature), MS—confirmed only by exact mass match, isotopic pattern and further evaluated with in silico prediction tools (MSC score), SI—semi-identified. Underlined are fragments that were the most abundant at each collision energy.
