# Supplementary material for: HPLC-DAD-ESI-QTOF-MS Determination of Bioactive Compounds and Antioxidant Activity Comparison of the Hydroalcoholic and Water Extracts from Two Helichrysum italicum Species
Source: Metabolites. 2020 Oct 12;10(10):403. doi: 10.3390/metabo10100403 (PMC7600872; doi:10.3390/metabo10100403)
Supplement: Supplementary file 1 [file metabolites-10-00403-s001.pdf]

SUPPLEMENTARY MATERIAL

HPLC-DAD-ESI-QTOF-MS Determination of Bioactive Compounds and Antioxidant Activity Comparison of the Hydroalcoholic and Water Extracts from two *Helichrysum Italicum* Species

Katja Kramberger [1,2], Darja Barlič-Maganja [1], Dunja Bandelj [3], Alenka Baruca Arbeiter [3], Kelly Peeters [4,5], Zala Jenko Pražnikar [1,*] and Ana Miklavčič Višnjevec [3,*]

[1] Faculty of Health Sciences, University of Primorska, 6310 Izola, Slovenia; katja.kramberger@fvz.upr.si, darja.maganja@fvz.upr.si, zala.praznikar@upr.si

[2] Faculty of Medicine, University of Ljubljana, 1000 Ljubljana, Slovenia; katja.kramberger@fvz.upr.si

[3] Faculty of Mathematics, Natural Sciences and Information Technologies, University of Primorska, 6000 Koper, Slovenia; dunja.bandelj@famnit.upr.si, alenka.arbeiter@upr.si, ana.miklavcic@famnit.upr.si

[4] InnoRenew Renewable Materials and Healthy Environments Research and Innovation Centre of Excellence, 6310 Izola, Slovenia; kelly.peeters@innorenew.eu

[5] Andrej Marušič Institute, University of Primorska, 6000 Koper, Slovenia; kelly.peeters@innorenew.eu

* Correspondence: ana.miklavcic@famnit.upr.si, zala.praznikar@upr.si; Tel.: +386-05-662-6469 (Z.J.P.)

Supplementary material
Kramberger et al.

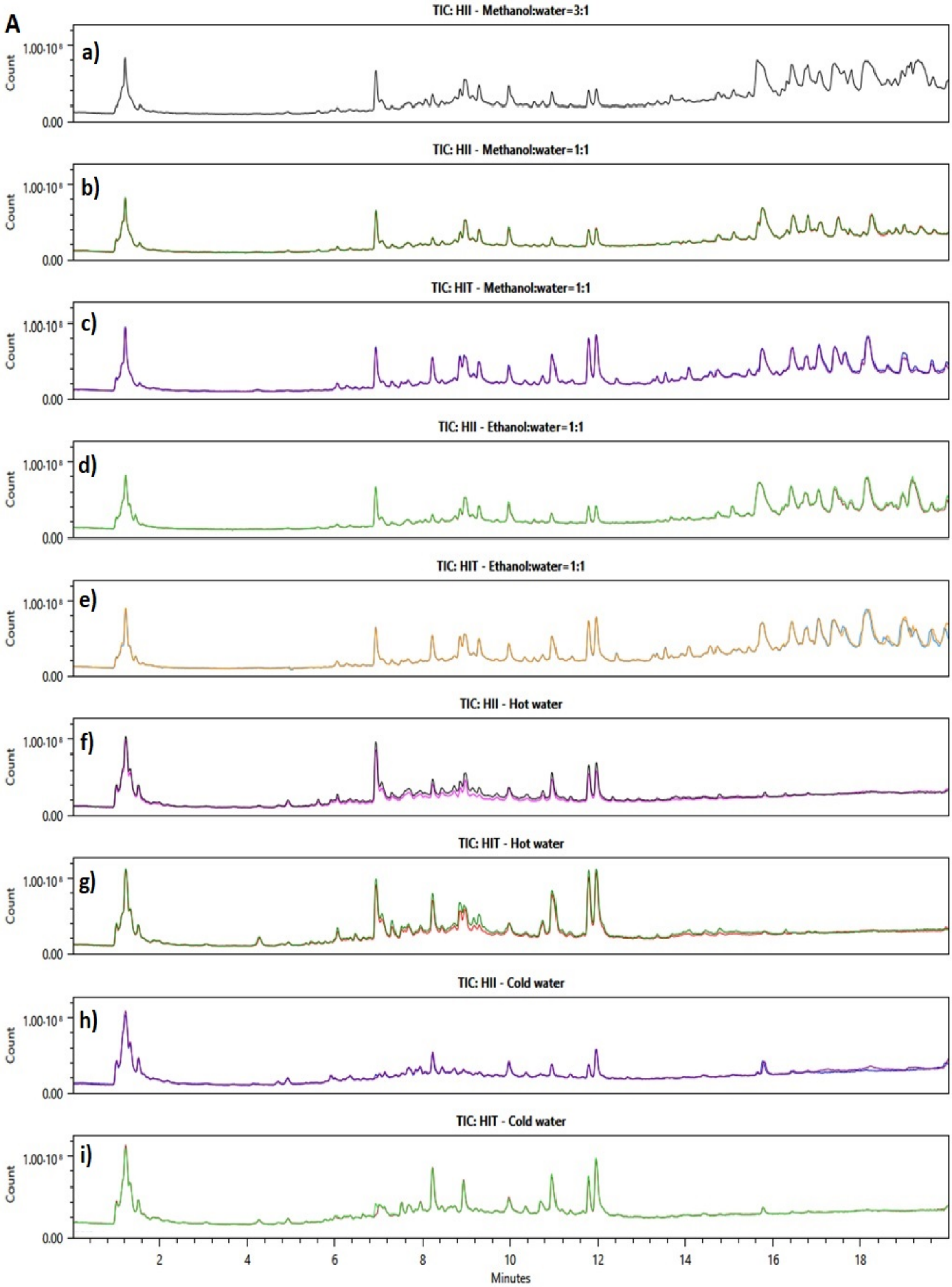

Supplementary material
Kramberger et al.

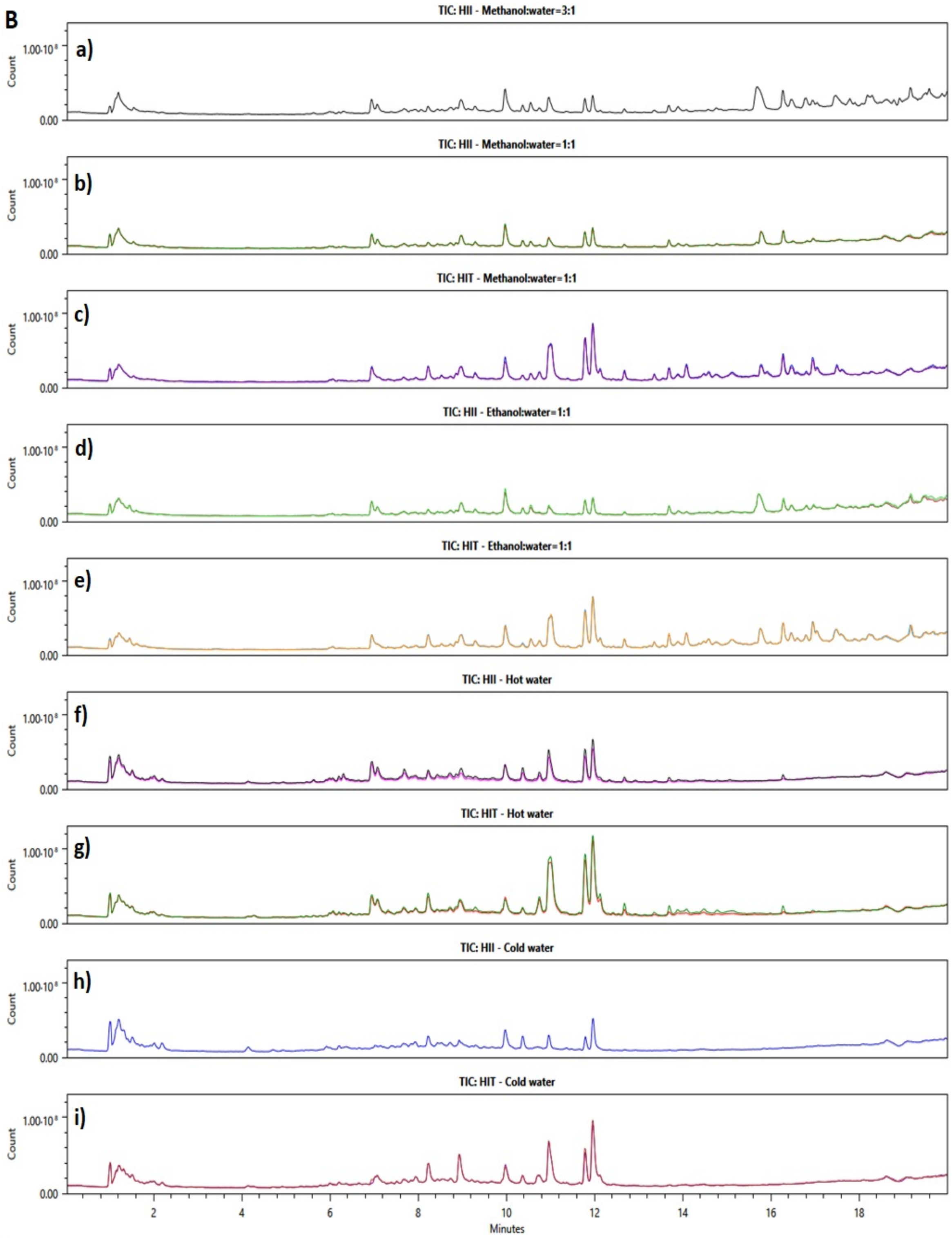

Figure S1. Total ion chromatograms of the tested samples gathered in negative (**A**) and positive (**B**) ESI mode. Samples are numbered accordingly: (a) H. italicum ssp. italicum (HII) methanol:water extract in ratio 3:1 and (b) HII methanol:water extract in equal ratios, (c) H. italicum ssp. tyrrhenicum (HIT) methanol:water extract in equal ratios, (d) and (e) HII and HIT ethanol:water extracts in equal ratios, (f) and (g) HII and HIT hot water extracts, (h) and (i) cold water extracts.

Supplementary material
Kramberger et al.

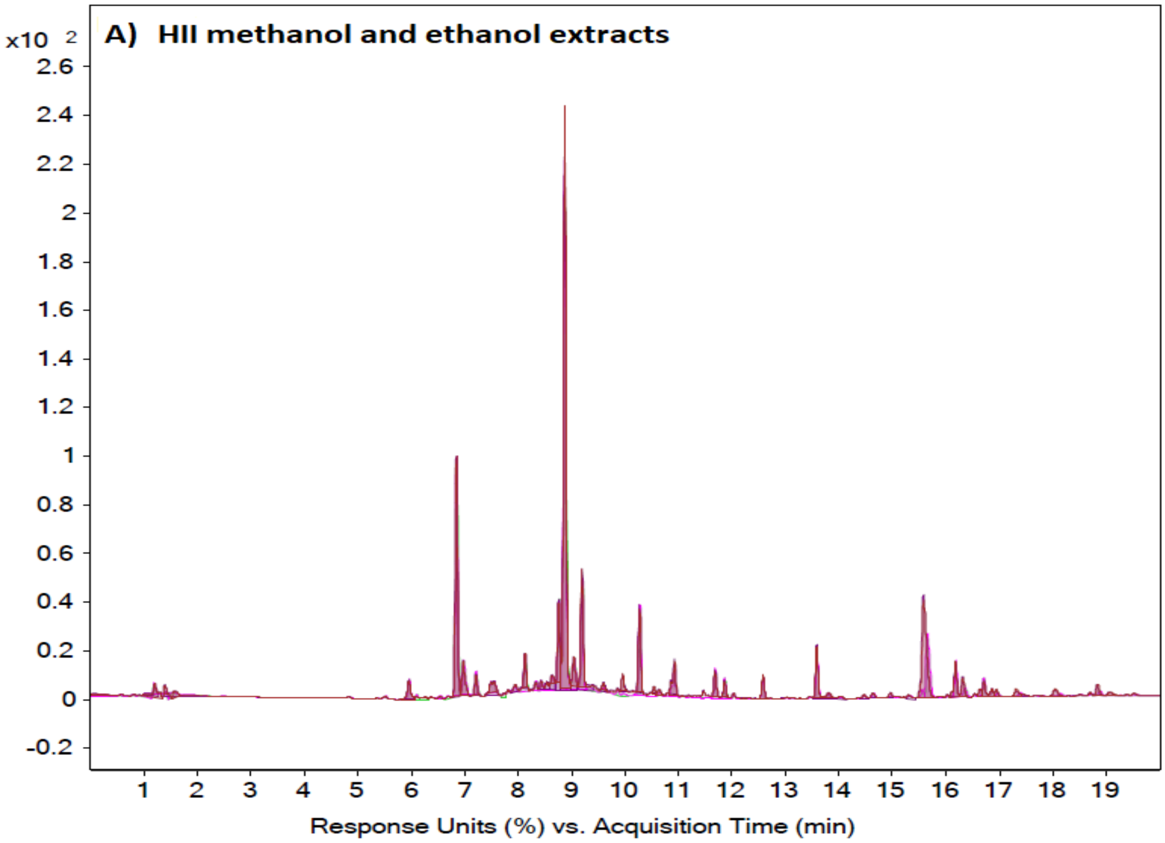

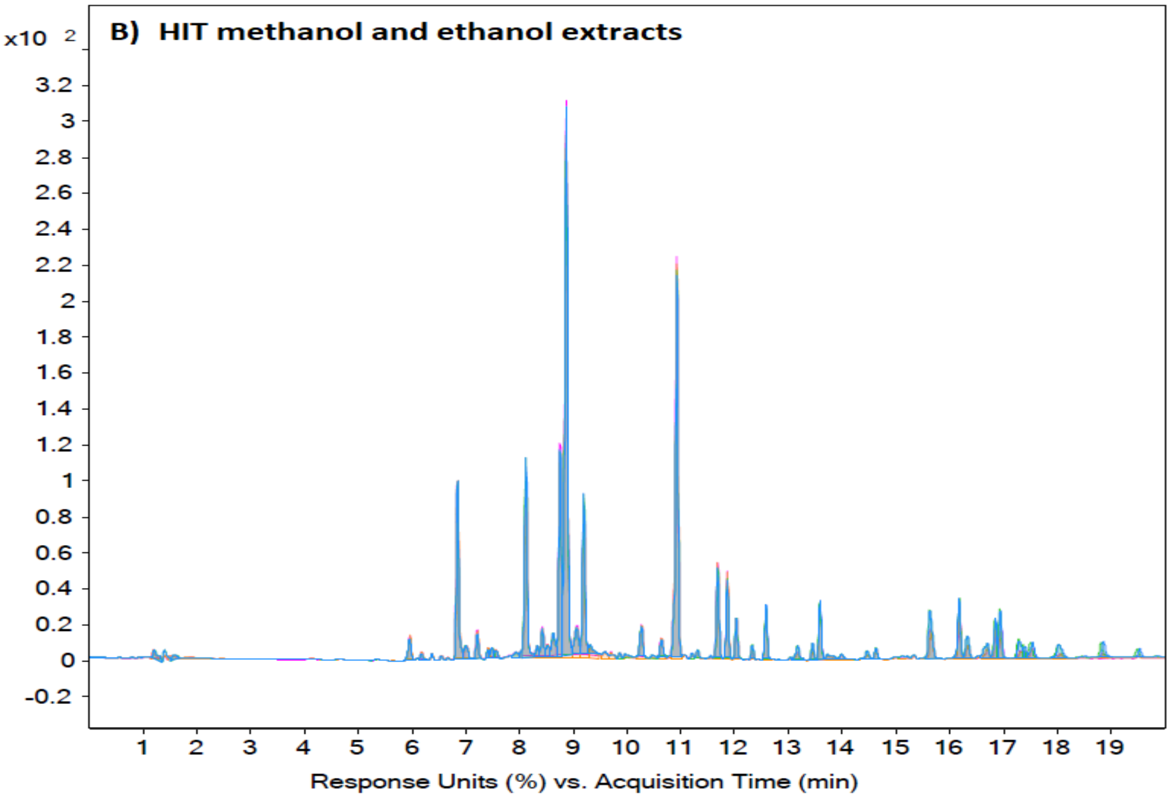

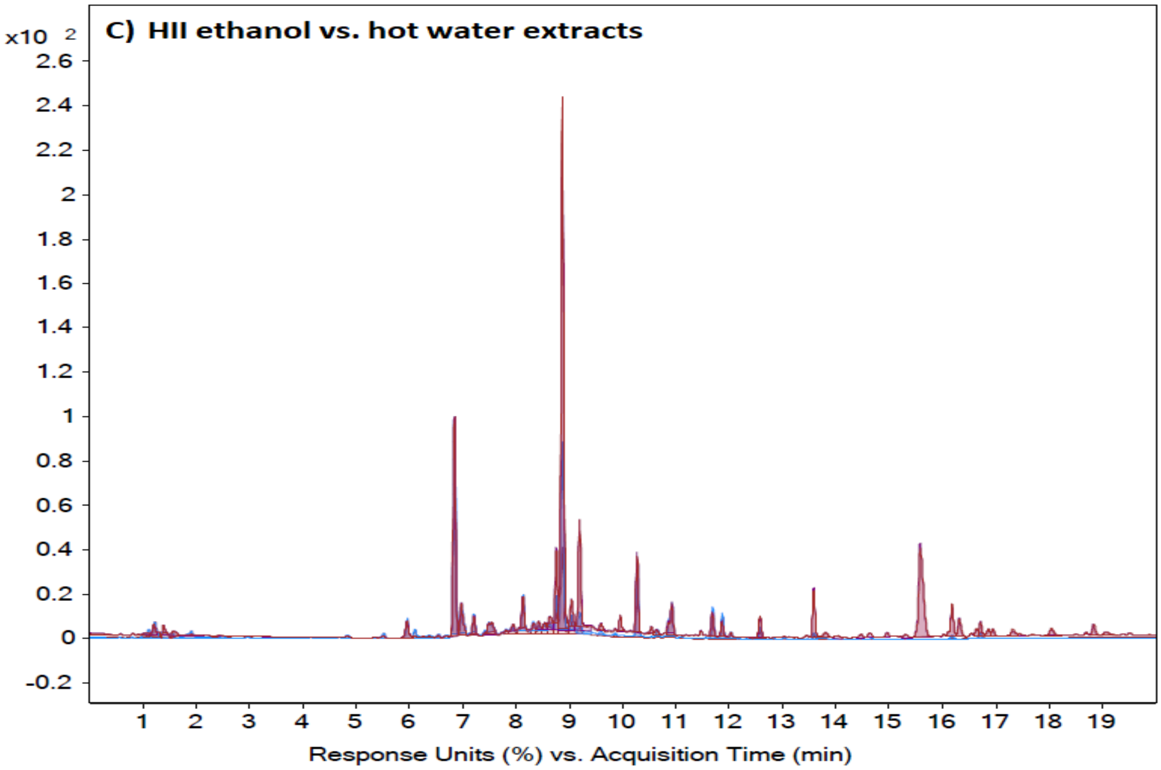
C) HII ethanol vs. hot water extracts
x10 2
2.6
2.4
2.2
2
1.8
1.6
1.4
1.2
1
0.8
0.6
0.4
0.2
0
-0.2
1 2 3 4 5 6 7 8 9 10 11 12 13 14 15 16 17 18 19
Response Units (%) vs. Acquisition Time (min)

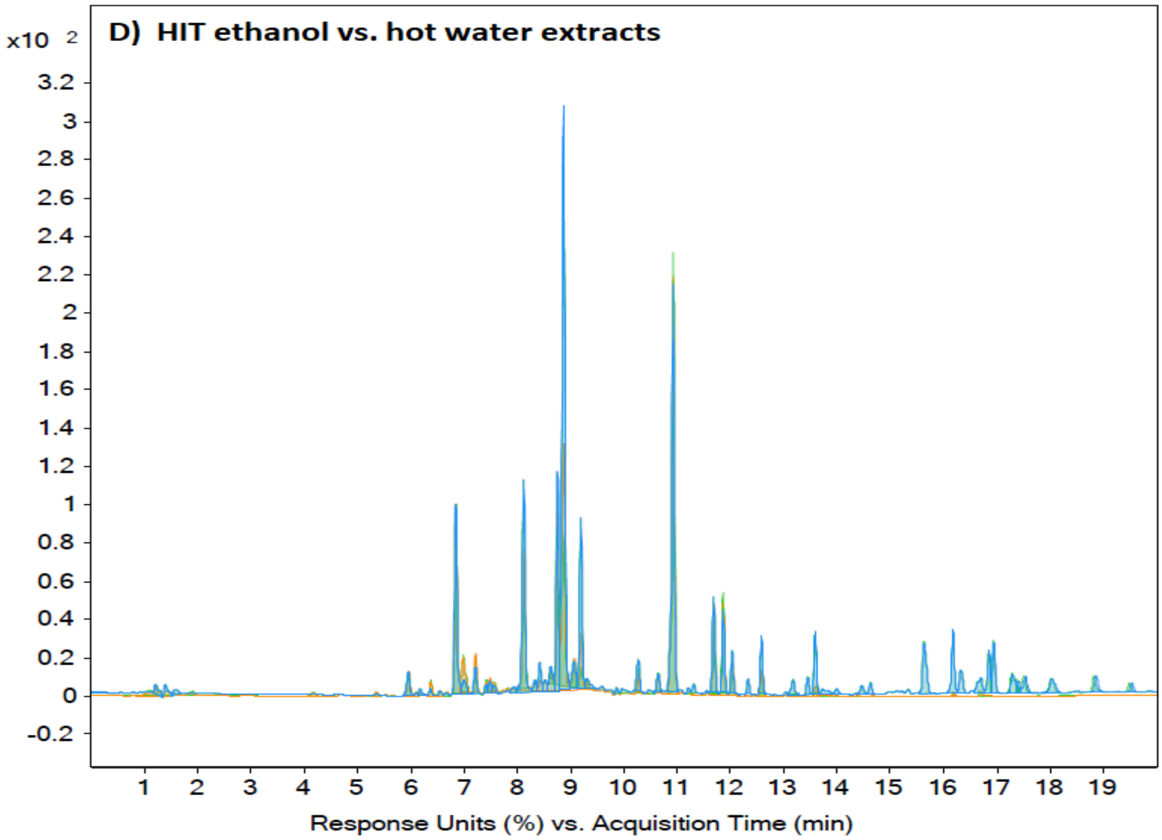
D) HIT ethanol vs. hot water extracts
x10 2
3.2
3
2.8
2.6
2.4
2.2
2
1.8
1.6
1.4
1.2
1
0.8
0.6
0.4
0.2
0
-0.2
1 2 3 4 5 6 7 8 9 10 11 12 13 14 15 16 17 18 19
Response Units (%) vs. Acquisition Time (min)

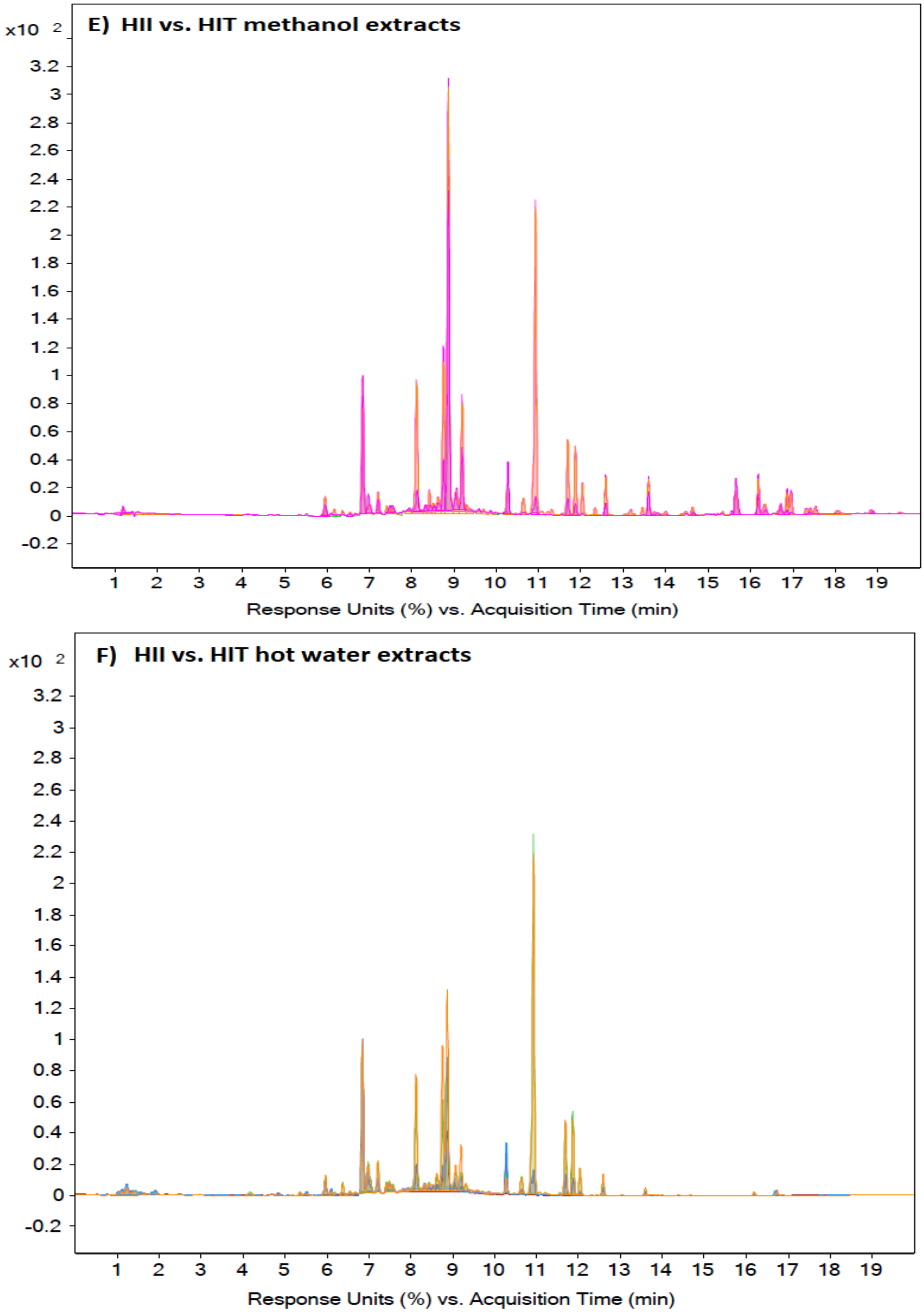

Figure S2. DAD chromatograms at 280 nm of the H. italicum samples overlaid accordingly: (A) and (B) representing differences in chemical profile between methanol and ethanol extracts of H. italicum ssp. italicum (HII) and H. italicum ssp. tyrrhenicum (HIT), respectively. (C) and (D) representing differences in ethanol versus hot water extracts of the HII and HIT, respectively, (E) and (F) representing differences between HII versus HIT methanol and hot water extracts, respectively.

Table S1. Results of the semi-quantitative analysis for the detected compounds in all tested samples. Values were calculated based on areas of extracted ion chromatograms (EICs) and corrected for dilution factor during sample preparation.

No. [1]	RT (min)	Compound class/ Name of the compound or tentative ID	Methanol:water extract		Ethanol:water extract		Hot water extract		Cold water extract	
			HII	*HIT*	*HII*	*HIT*	*HII*	*HIT*	*HII*	*HIT*
		**Hydroxycinnamic acids**								
Free hydroxycinnamic acids and their glycosides										
		Caffeic acid and its derivatives								
9	6.24	Caffeic acid O-hexoside 1	7582	1981	n.d.	n.d.	n.d.	2897	12448	18914
14	6.88	Caffeic acid O-hexoside 2	13180	5748	14632	5430	10678	6467	14509	18725
16	7.05	Caffeic acid O-hexoside 3	13915	9704	15118	8836	9485	14905	49056	81669
21	7.3	Caffeic acid	59286	128100	84254	108668	65563	77201	215217	104112
57	9.3	Caffeic acid O-hexoside derivative	n.q.	17201	n.d.	n.q	n.q	n.d.	57806	n.d.
		Total caffeic acid and its derivatives	93962	162735	114004	122934	85726	101471	349036	223420
		Other hydroxycinnamic acids and their derivatives								
19	7.15	Coumaric acid hexoside 1	34060	38692	30615	34477	21455	52757	234986	444668
24	7.57	Coumaric acid hexoside 2	11581	n.d.	8999	n.d.	n.d.	n.d.	n.d.	n.d.
40	8.63	p-Coumaric acid	n.d.	23175	n.d.	15994	n.d.	3360	20595	40196
65	9.8	Ferulic acid	72827	126813	n.d.	n.d.	n.d.	n.d.	n.d.	n.d.
		Total other hydroxycinnamic acids	118468	188680	39613	50471	21455	56117	255581	484864
Total free hydroxycinnamic acids and their glycosides			212430	351415	153617	173405	107181	157587	604616	708284
Monoesters with hydroxycinnamic acids										
		CQAs								
4	5.55	CQA-glucoside: 3-O-(4'-caffeoyl glucosyl) quinic acid	n.d.	n.d.	n.d.	n.d.	n.d.	2941	16964	38953
6	6.01	CQA-glucoside: 5-O-(4'-caffeoyl glucosyl) quinic acid	7600	26602	7647	24042	8863	19148	101058	240447
7	6.06	Caffeoylquinic acid isomer: 3-O-CQA	141562	253531	151345	215064	133048	193301	44170	154985
12	6.64	CQA-glucoside: 5-O-(3'-caffeoyl glucosyl) quinic acid	17632	52990	18749	47538	20860	39077	82112	328932
15	6.95	Chlorogenic acid (5-O-CQA)	924240	976417	976779	933696	486566	491833	187275	573661
17	7.1	Caffeoylquinic acid isomer: 4-O-CQA	168547	229632	172239	201408	128790	192436	43153	111877
25	7.6	Caffeoylquinic acid isomer: cis-chlorogenic acid	81859	25710	86551	14643	78749	22374	n.d.	n.d.

		Total CQAs	1341439	1564882	1413311	1436390	856875	961110	474732	1448855
		Other monoesters								
26	7.66	Coumaroylquinic acid	n.d.	6399	5193	5633	4662	4918	21094	20384
31	8.04	5-O-Feruloylquinic acid	34421	11713	39780	11174	31863	11256	47354	n.d.
34	8.25	5-O-Coumaroylquinic acid	11434	1092	13720	n.d.	10853	n.d.	55742	n.d.
63, 67	9.69	3-Feruloyl-5-caffeoylquinic acid, 4-feruloyl-5-caffeoylquinic acid	44049	20345	45238	n.d.	n.d.	n.d.	n.d.	n.d.
		Total other monoesters	89904	39548	103930	16808	47378	16174	124190	20384
Total monoesters with hydroxycinnamic acids			1431342	1604430	1517241	1453198	904254	977284	598922	1469240
DiCQAs										
22	7.32	Dicaffeoylhexaric acid	116891	197892	87487	147363	79545	230462	n.d.	135620
36	8.44	Dicaffeoylquinic acid glycoside	n.d.	n.d.	n.d.	n.d.	n.d.	n.d.	n.d.	n.d.
46, 49, 51	8.96	Dicaffeoyl quinic acids: 3,4, 3,5 and 1,5-diCQA	2390790	3063876	2510245	2897507	675315	950404	n.d.	n.d.
56	9.29	Dicaffeoyl quinic acid: 4,5- diCQA	859001	1401124	959653	1351789	145588	345215	n.d.	n.d.
59	9.41	Methoxyoxayl-dicaffeoylquinic acid	n.d.	36007	9402	42159	4445	37523	n.d.	n.d.
38	8.49	Caffeoyl derivative	27347	22116	25933	17532	34797	20396	n.d.	n.d.
Total DiCQAs			3394029	4721015	3592719	4456350	939690	1584000	0	135620
TriCQAs										
52	9.15	Tricaffeoylhexaric acid	158777	746413	146070	679956	72330	530427	n.d.	n.d.
72	10.34	Tricaffeoylquinic acid	7188	67703	18331	100167	n.d.	n.d.	n.d.	n.d.
Total triCQAs			165965	814116	164401	780123	72330	530427	n.d.	n.d.
**Total hydroxycinnamic acids**			5203767	7490976	5427977	6863076	2023456	3249299	1203538	2313143
			**Hydroxybenzoic acids and their glycosides**							
Monohydroxybenzoic acids										
10	6.28	Hydroxybenzoic acid derivative	n.d.	20612	n.d.	14501	n.d.	n.d.	n.d.	n.d.
11	6.53	4-Hydroxybenzoic acid	7753	n.d.	10592	n.d.	5597	4310	157963	55260
13	6.82	Hydroxybenzoic acid hexoside	18443	13490	16915	12371	12852	22490	70113	160878
62	9.66	Salicylic acid	n.d.	19272	3645	14829	2448	5543	54598	72373
Total monohydroxybenzoic acids			26196	53375	31152	41701	20897	32343	282673	288511
Dihydroxybenzoic acids										
3	4.92	Protocatechuic acid O-hexoside	52012	77243	46166	105577	68801	681256	579406	1226
8	6.13	Vanillic acid derivative 1	14022	8343	14037	6982	15404	12838	76618	77167
18	7.12	Vanillic acid O-hexoside	6688	10825	6809	10290	4513	8069	n.d.	15524
23	7.51	2,4-Dihydroxybenzoic acid	n.d.	n.d.	n.d.	n.d.	n.d.	n.d.	n.d.	47169
27	7.67	Vanillic acid derivative 2	3298	n.d.	n.d.	n.d.	n.d.	n.d.	n.d.	n.d.

29	7.87	Vanillic acid derivative 3	12702	n.d.	5993	n.d.	5698	4206	n.d.	n.d.
35	8.33	Dihydroxybenzoic acid derivative	n.d.	n.d.	n.d.	n.d.	n.d.	n.d.	7792	3439
Total dihydroxybenzoicacid derivatives			88722	96411	73005	122849	94417	706369	663816	144524
Trihydroxybenzoic acids										
1	2.97	Gallic acid glucoside	n.d.	9396	n.d.	n.d.	n.d.	9933	n.d.	26563
		Total trihydroxybenzoic acids	n.d.	9396	n.d.	n.d.	n.d.	9933	n.d.	26563
**Total hydroxybenzoic acids**			114918	159182	104157	164550	115314	748645	946489	459598
						**Flavonoids**				
Flavonols										
		Quercetin and its derivatives								
28	7.7	Quercetin diglycoside	n.d.	2357	n.d.	1750	n.d.	2578	n.d.	n.d.
33	8.21	Quercetin hexoside 1	n.d.	470654	n.d.	619469	n.d.	154633	n.d.	n.d.
41, 45	8.77	Quercetin hexoside 2 and 3	97091	13985	126391	9570	38874	n.d.	n.d.	n.d.
43	8.71	Quercetin malonylhexoside 1	n.d.	92499	n.d.	105332	n.d.	101790	n.d.	n.d.
50	8.96	Quercetin malonylhexoside 2	38365	n.d.	43941	n.d.	47617	n.d.	n.d.	n.d.
64	9.73	Quercetin hexoside 4	n.d.	n.d.	32531	n.d.	n.d.	n.d.	n.d.	n.d.
68	10.05	Quercetin coumaroylhexoside 1	n.d.	8557	254457	44547	n.d.	n.d.	n.d.	n.d.
70	10.24	Quercetin coumaroylhexoside 2	n.d.	n.d.	43492	4499	n.d.	n.d.	n.d.	n.d.
73	10.57	Quercetin	n.d.	n.d.	8899	n.d.	n.d.	n.d.	n.d.	n.d.
85	11.8	Quercetin dimethyl ether	n.d.	n.d.	n.d.	n.d.	n.d.	n.d.	n.d.	n.d.
48	8.94	Isorhamnetin hexoside 1	11317	11807	7620	10914	9951	n.d.	n.d.	n.d.
58	9.4	Isorhamnetin hexoside 2	28890	n.d.	39387	n.d.	n.d.	1085	n.d.	n.d.
77	10.98	Isorhamnetin 1	n.d.	5913	n.d.	36194	n.d.	n.d.	n.d.	n.d.
83	11.6	Isorhamnetin 2	n.d.	n.d.	n.d.	n.d.	n.d.	n.d.	n.d.	n.d.
		Total quercetin and its derivatives	175663	605770	556718	832273	96442	260087	n.d.	n.d.
		Myricetin derivatives								
32	8.06	Myricetin glucoside	n.d.	24045	n.d.	n.d.	n.d.	n.d.	n.d.	n.d.
39	8.56	Myricetin malonyl hexoside	13562	n.d.	23496	3840	16306	n.d.	n.d.	n.d.
47	8.89	Myricetin acetylglycoside	2477	8468	n.d.	n.d.	5429	11080	32277	79841
		Total myricetin derivatives	16039	32513	23496	3840	21735	11080	32277	79841
		Kaempferol and its derivatives								
30	8.03	Kaempferol diglycoside	4046	12065	n.d.	8843	n.d.	n.d.	n.d.	n.d.
54	9.26	Kaempferol glycoside	n.d.	10036	n.d.	n.d.	n.d.	n.d.	n.d.	n.d.
61	9.52	Kaempferol acetylglycoside	1266	71111	n.d.	n.d.	1226	n.d.	4674	n.d.
75	10.56	Tiliroside	n.d.	5338	34044	n.d.	n.d.	n.d.	n.d.	n.d.

81	11.44	Kaempferol	n.d.	n.d.	n.d.	n.d.	n.d.	n.d.	n.d.	n.d.
84	11.85	Isokaempferide 1	n.d.	n.d.	5820	1239	n.d.	n.d.	n.d.	n.d.
87	12.35	Isokaempferide 2	n.d.	1002	n.d.	1748	n.d.	n.d.	n.d.	n.d.
		Total kaempferol and its derivatives	5313	99551	39864	11830	1226	n.d.	4674	n.d.
		Other flavonols								
90	13.87	Gnaphaliin A	4476	n.d.	6360	5219	n.d.	n.d.	n.d.	n.d.
91	13.94	Galangin methyl ether	17228	28308	92224	74534	n.d.	n.d.	n.d.	n.d.
37	8.4	Herbacetin methyl ether	n.d.	n.d.	n.d.	n.d.	n.d.	n.d.	10349	52117
55	9.28	Herbacetin	n.d.	14877	n.d.	34484	n.d.	n.d.	n.d.	n.d.
		Total other flavonols	21704	43185	98584	114236	n.d.	n.d.	10349	52117
Total flavonols			415733	1518854	1338738	1810122	238806	542334	84250	211799
Flavanones										
60	9.51	Eriodictyol hexoside	6781	2077	7321	7124	n.d.	n.d.	n.d.	n.d.
71	10.32	Eriodictyol	n.d.	11919	3242	13556	n.d.	n.d.	n.d.	n.d.
74	10.55	Pinocembrin derivative	n.d.	18202	n.d.	17765	n.d.	14043	n.d.	n.d.
82	11.47	Pinocembrin isomer	n.d.	6054	n.d.	5932	n.d.	7174	n.d.	17985
89	13.28	Pinocembrin	5634	302697	14414	459714	n.d.	n.d.	n.d.	n.d.
78	11.13	Naringenin	n.d.	15688	n.d.	23069	n.d.	n.d.	n.d.	n.d.
80	11.41	Naringenin isomer	6953	372489	10458	393396	n.d.	11913	n.d.	n.d.
Total flavanones			19367	729127	35436	920555	n.d.	33129	n.d.	17985
Flavones										
76	10.6	Luteolin	n.d.	n.d.	n.d.	40750	n.d.	n.d.	n.d.	n.d.
		Total flavones	n.d.	n.d.	n.d.	40750	n.d.	n.d.	n.d.	n.d.
**Total flavonoids**			891903	4539146	2846930	5616341	477612	1150926	178849	511685
					**Coumarins**					
20	7.25	Esculetin	29952	43739	32105	43468	23254	25597	n.d.	n.d.
42	8.68	Scopoletin	6987	n.d.	8521	n.d.	2748	n.d.	21382	n.d.
**Total coumarins**			36940	43739	40626	43468	26002	25597	21382	n.d.
					**Arzanol derivatives and other pyrones**					
Pyrones										
86	11.96	Micropyrone	1735894	3907602	1665340	3484926	1151710	2124196	5468528	9153190
94	16.28	Helipyrone	77208	146326	81833	159131	10619	14422	n.d.	n.d.
95	16.63	Italipyrone 1	338814	386731	299746	317227	n.d.	n.d.	n.d.	n.d.
100	18.8	Italipyrone 2	416750	49524	400206	133899	n.d.	n.d.	30734	10087
Total pyrones			2568665	4490184	2447125	4095183	1162329	2138619	5499262	9163277
Arzanol and its derivatives										
92	15.64	Heliarzanol 1	1155582	481583	968377	479289	14157	9179	386320	52458

93	15.77	Arzanol	4463785	3991846	5601354	4582094	110240	77697	2762884	1243023
96	16.46,16.76	3-O-Methylarzanol	n.d.	4871748	n.d.	6163727	25016	27347	590040	313884
97	17.45	Arzanol derivative 1	57421	79219	11854	32733	n.d.	n.d.	n.d.	n.d.
98	17.93	Arzanol isomer	47972	10137	50881	27677	n.d.	n.d.	n.d.	n.d.
99	17.97	Heliarzanol 2	n.q.	n.d.	n.q.	n.q.	n.d.	n.d.	n.d.	n.d.
Total arzanol derivatives			5724759	9434533	6632466	11285520	149413	114223	3739244	1609366
**Total arzanol derivatives and other pyrones**			8293424	13924716	9079591	15380703	1311742	2252842	9238506	10772643
**Others (isobenzofuranones, neolignans, acetophenones, tremetones)**										
		Isobenzofuranones								
2	4.25	Hydroxyphthalide glucoside 1	10624	39688	n.d.	36971	12974	66262	n.d.	147843
5	5.63	Hydroxyphthalide glucoside 2	91049	21672	86836	31855	102779	56079	11931	127614
		Total isobenzofuranones	101674	61361	86836	68826	115753	122341	11931	275457
		Neolignans								
44	8.73	Dihydrodehyrodiconiferyl glucoside derivative	174042	108521	179881	96202	96328	79040	470171	428903
53	9.23	Dihydrodehyrodiconiferyl glucoside derivative	39318	32664	n.d.	26385	20207	164423	157639	191993
		Total neolignans	213360	141185	179881	122587	116535	243463	627810	620896
		Acetophenones								
66, 69	10.08	4-hydroxy-3-(2-hydroxy-3-isopentenyl) acetophenone 1 and 2	11950	29672	11274	23884	3952	8124	19231	34327
88	13.23	3-Prenyl-4-hydroxyacetophenone	19659	93505	25868	97559	n.d.	3647	n.d.	n.d.
		Total acetophenones	31609	123177	37142	121443	3952	11771	19231	34327
		Tremetones								
79	11.18	Gnaphaliol glucopyranoside	17762	93102	16254	68595	8122	47072	56069	257825
		Total tremetones	17762	93102	16254	68595	8122	47072	56069	257825
**Total other phenolic compounds**			364405	418825	320113	381451	244362	424648	715041	1188505
**TOTAL IDENTIFIED PHENOLIC COMPOUNDS**			14905358	26576584	17819394	28449589	4198487	7851956	12303805	15245573

[1] – Compounds are numbered according to the order of elution from the column.
